# Onset ages of cerebrovascular disease and amyloid and effects on cognition in risk-enriched cohorts

**DOI:** 10.1093/braincomms/fcaf158

**Published:** 2025-04-19

**Authors:** Lianlian Du, Elizabeth M Planalp, Tobey J Betthauser, Erin M Jonaitis, Bruce P Hermann, Leonardo A Rivera-Rivera, Karly A Cody, Nathaniel A Chin, Robert V Cadman, Kevin M Johnson, Aaron Field, Howard A Rowley, Kimberly D Mueller, Sanjay Asthana, Laura Eisenmenger, Bradley T Christian, Sterling C Johnson, Rebecca E Langhough

**Affiliations:** Wisconsin Alzheimer’s Institute, University of Wisconsin-Madison School of Medicine and Public Health, Madison, WI 53792, USA; Department of Medicine, University of Wisconsin-Madison School of Medicine and Public Health, Madison, WI 53792, USA; Rush Alzheimer’s Disease Center, Rush University Medical Center, Chicago, IL 60612, USA; Department of Neurological Sciences, Rush University Medical Center, Chicago, IL 60612, USA; Wisconsin Alzheimer’s Institute, University of Wisconsin-Madison School of Medicine and Public Health, Madison, WI 53792, USA; Department of Medicine, University of Wisconsin-Madison School of Medicine and Public Health, Madison, WI 53792, USA; Wisconsin Alzheimer’s Disease Research Center, Department of Medicine, University of Wisconsin-Madison School of Medicine and Public Health, Madison, WI 53792, USA; Department of Medicine, University of Wisconsin-Madison School of Medicine and Public Health, Madison, WI 53792, USA; Wisconsin Alzheimer’s Disease Research Center, Department of Medicine, University of Wisconsin-Madison School of Medicine and Public Health, Madison, WI 53792, USA; Wisconsin Alzheimer’s Institute, University of Wisconsin-Madison School of Medicine and Public Health, Madison, WI 53792, USA; Department of Medicine, University of Wisconsin-Madison School of Medicine and Public Health, Madison, WI 53792, USA; Wisconsin Alzheimer’s Disease Research Center, Department of Medicine, University of Wisconsin-Madison School of Medicine and Public Health, Madison, WI 53792, USA; Wisconsin Alzheimer’s Institute, University of Wisconsin-Madison School of Medicine and Public Health, Madison, WI 53792, USA; Department of Neurology, University of Wisconsin-Madison School of Medicine and Public Health, Madison, WI 53792, USA; Department of Medicine, University of Wisconsin-Madison School of Medicine and Public Health, Madison, WI 53792, USA; Wisconsin Alzheimer’s Disease Research Center, Department of Medicine, University of Wisconsin-Madison School of Medicine and Public Health, Madison, WI 53792, USA; Department of Medical Physics, University of Wisconsin-Madison School of Medicine and Public Health, Madison, WI 53792, USA; Wisconsin Alzheimer’s Disease Research Center, Department of Medicine, University of Wisconsin-Madison School of Medicine and Public Health, Madison, WI 53792, USA; Department of Neurology and Neurological Sciences, Stanford University, Palo Alto, CA 94304, USA; Department of Medicine, University of Wisconsin-Madison School of Medicine and Public Health, Madison, WI 53792, USA; Wisconsin Alzheimer’s Disease Research Center, Department of Medicine, University of Wisconsin-Madison School of Medicine and Public Health, Madison, WI 53792, USA; Department of Medicine, University of Wisconsin-Madison School of Medicine and Public Health, Madison, WI 53792, USA; Wisconsin Alzheimer’s Disease Research Center, Department of Medicine, University of Wisconsin-Madison School of Medicine and Public Health, Madison, WI 53792, USA; Department of Medical Physics, University of Wisconsin-Madison School of Medicine and Public Health, Madison, WI 53792, USA; Department of Radiology, University of Wisconsin School of Medicine and Public Health, Madison, WI 53792, USA; Wisconsin Alzheimer’s Disease Research Center, Department of Medicine, University of Wisconsin-Madison School of Medicine and Public Health, Madison, WI 53792, USA; Department of Radiology, University of Wisconsin School of Medicine and Public Health, Madison, WI 53792, USA; Wisconsin Alzheimer’s Disease Research Center, Department of Medicine, University of Wisconsin-Madison School of Medicine and Public Health, Madison, WI 53792, USA; Department of Radiology, University of Wisconsin School of Medicine and Public Health, Madison, WI 53792, USA; Wisconsin Alzheimer’s Institute, University of Wisconsin-Madison School of Medicine and Public Health, Madison, WI 53792, USA; Department of Medicine, University of Wisconsin-Madison School of Medicine and Public Health, Madison, WI 53792, USA; Wisconsin Alzheimer’s Disease Research Center, Department of Medicine, University of Wisconsin-Madison School of Medicine and Public Health, Madison, WI 53792, USA; Department of Communication Sciences and Disorders, University of Wisconsin-Madison, Madison, WI 53792, USA; Department of Medicine, University of Wisconsin-Madison School of Medicine and Public Health, Madison, WI 53792, USA; Wisconsin Alzheimer’s Disease Research Center, Department of Medicine, University of Wisconsin-Madison School of Medicine and Public Health, Madison, WI 53792, USA; Department of Radiology, University of Wisconsin School of Medicine and Public Health, Madison, WI 53792, USA; Department of Medical Physics, University of Wisconsin-Madison School of Medicine and Public Health, Madison, WI 53792, USA; Wisconsin Alzheimer’s Institute, University of Wisconsin-Madison School of Medicine and Public Health, Madison, WI 53792, USA; Department of Medicine, University of Wisconsin-Madison School of Medicine and Public Health, Madison, WI 53792, USA; Wisconsin Alzheimer’s Disease Research Center, Department of Medicine, University of Wisconsin-Madison School of Medicine and Public Health, Madison, WI 53792, USA; Wisconsin Alzheimer’s Institute, University of Wisconsin-Madison School of Medicine and Public Health, Madison, WI 53792, USA; Department of Medicine, University of Wisconsin-Madison School of Medicine and Public Health, Madison, WI 53792, USA; Wisconsin Alzheimer’s Disease Research Center, Department of Medicine, University of Wisconsin-Madison School of Medicine and Public Health, Madison, WI 53792, USA

**Keywords:** Alzheimer’s disease, white matter hyperintensities, positron emission tomography, time operationalization, preclinical cognitive decline

## Abstract

The temporal relationship between cerebrovascular disease (V), indicated by white matter hyperintensities, and beta-amyloid (A) in Alzheimer’s disease remains unclear, prompting speculation about their potential interdependence. Longitudinal data were employed to estimate onset ages and corresponding disease chronicity for A and V (where disease chronicity is calculated as age at measurement minus estimated age of biomarker abnormality onset). In a large, predominantly cognitively unimpaired dataset (*n* = 877, ages 43–93 years), a V+ threshold was identified, and Sampled Iterative Local Approximation (SILA) was utilized to illustrate the predictable accumulation trajectory of V post-onset. Investigating the temporal association between A and V onset ages and accumulation trajectories in preclinical years, four operationalizations of time were examined across two initially cognitively unimpaired samples (*n* = 240 primary sample from Wisconsin Registry for Alzheimer’s Prevention; *n* = 123 replication sample from Wisconsin Alzheimer’s Disease Research Center): (i) chronological age, (ii) estimated V+ chronicity, (iii) years since baseline scan, and (iv) estimated A+ chronicity. Results indicated that while both diseases are age-related, their onsets and trajectories are independent of each other. In addition, results indicated that V and A accumulation trajectories were highly predictable relative to onset of positivity for each biomarker. Cognitive decline across multiple cognitive domains was fastest when both V and A were present based on last available amyloid PET and MRI scan, with greater A chronicity being a more salient predictor of cognitive decline in these samples.

## Teaser

Du *et al*. showed that the age-of-onset and disease duration of vascular ischaemic lesions (V) can be estimated. They found that the onset and trajectories of V and beta-amyloid (A) in Alzheimer's are independent. Cognitive decline is fastest when both are present, with A chronicity being a more significant predictor.

## Graphical Abstract

**Graphical Abstract:**
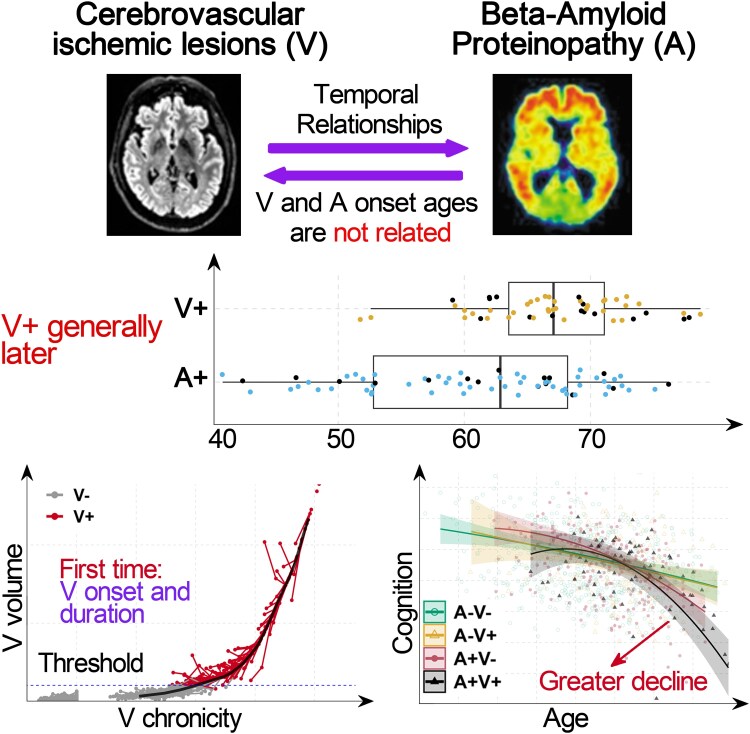

## Introduction

Alzheimer’s disease is characterized by physiological and cognitive changes that lead to functional decline.^1^ Well known in the Alzheimer’s disease literature, amyloid β plaques and neurofibrillary tau tangles are Alzheimer’s disease defining features^2^ which accumulate in the brain leading to progressive neurodegeneration and cognitive and functional decline.^3,4^ Cerebrovascular disease (CVD or V as used in this study) can also contribute to morphological brain changes associated with cognitive decline and is a primary cause of vascular dementia.^5-8^ In brain donors with autopsy-confirmed Alzheimer’s disease, comorbid CVD has been associated with accelerated functional and cognitive decline.^9^ White matter hyperintensities (WMH), when less common processes have been ruled out, are an indicator of presumed microvascular alterations and manifest as areas of increased signal on T2-weighted fluid attenuated inversion recovery (FLAIR) MRI images.^10^ Some previous work in both autosomal dominant and late-onset Alzheimer’s disease suggests that WMH may play a role in the evolution of Alzheimer’s disease and in particular to beta-amyloid progression.^11,12^ Indeed, amyloid-PET and WMH associations in older adults have been interpreted as related processes,^11,13,14^ though the research remains mixed^15,16^ with recent longitudinal work instead suggesting separate processes.^17^

It is likely that the few existing studies linking WMH, amyloid, and other Alzheimer’s disease related outcomes are limited. This could be due to the use of cross-sectional data or variability in T2-FLAIR protocols, especially when data is pulled from different cohorts. Additionally, differences in cohort composition, such as Alzheimer’s Disease Neuroimaging Initiative screening out CVD, and the use of statistical models that operationalize time in inconsistent ways, contribute to inconsistencies across studies, as described here. The three time-operationalizations that have been used to model outcome trajectories include observed age at assessment, time since baseline assessment (often covarying baseline age), and more recently, the chronicity of a potential predictor condition such as amyloid positivity. Most studies rely on a one-time operationalization model without comparisons with others, which may limit the interpretation of longitudinal changes in Alzheimer’s disease related biomarkers.^18-20^ For example, some research has found that initial WMH predicted steeper increases in amyloid-PET; however, conflicting results have been observed regarding the associations between changes in WMH and changes in amyloid-PET within the same study.^20^

Recently, our group and others^21-25^ have developed techniques for estimating an onset age for Alzheimer’s disease biomarker abnormality, enabling rescaling such that zero years indicates the point where biomarker positivity is reached. In this way, onset age as well as time with disease can be indicated on a biomarker chronicity scale describing the chronicity of disease from its earliest manifestation rather than at symptom onset. This time operationalization of amyloid burden has led to increased understanding of contributors to tau-PET abnormalities,^23,25^ preclinical cognitive decline,^23^ and progression to dementia.^21,24^ This invites application of these modelling techniques to other brain markers such as WMH. Recently, Cogswell *et al.*^17^ extended use of accelerated failure time modelling to WMH trajectories in a sample of >2400 individuals (mean (SD) age 74 (11)) to create an adjusted time scale based on WMH level. Interestingly, their longitudinal analyses did not find significant associations between Alzheimer’s disease related biomarkers of amyloid or tau and CVD markers such as WMH.

In this paper we applied the Sampled Iterative Local Approximation (SILA) method^21^ to longitudinal WMH data to align WMH development relative to years before or since WMH onset. We hypothesize that any dependencies between V and A onsets and trajectories would be better explained when anchored to their estimated biomarker onset ages and chronicities. We further surmise that *how* time is operationalized influences interpretation of V and A relationships in the progression of Alzheimer’s disease; thus, direct comparisons of the various options are needed, particularly in pre-clinical stages of cognitive decline. To that end, we first investigated WMH trajectories relative to age and SILA-aligned WMH chronicities to characterize typical WMH changes in V− versus V+ subgroups (Aim 1). We then considered longitudinal WMH, amyloid-PET and cognitive trajectories in a sample of baseline cognitively unimpaired (CU) individuals from the Wisconsin Registry of Alzheimer’s Prevention (WRAP) who had also completed at least one amyloid PET scan. Specifically, in Aim 2a, we compared models of WMH trajectories using four operationalizations of time: (i) chronological age, (ii) estimated V+ chronicity, (iii) years since baseline scan and (iv) estimated A+ chronicity. In Aim 2b, we performed parallel analyses of longitudinal amyloid-PET trajectories. In Aim 3, we examined how the presence of A+ and V+ contributes to longitudinal decline across multiple cognitive domains. Finally, Aim 4 gauged the robustness of our findings by repeating the Aim 2 and 3 analyses in a parallel sample from the Wisconsin Alzheimer's Disease Research Center (WADRC) cohort.

## Materials and methods

### Participants

Participants for Aim 1 analyses included 272 WRAP and 605 WADRC who had completed at least one T1-weighted and T2-weighted FLAIR MRI scan and had estimated V+ onset ages using SILA modelling from a May 2022 data freeze.

Aims 2 and 3 selected participants from WRAP, a longitudinal cohort study enriched for individuals at risk of dementia due to a parental Alzheimer’s history.^26^ The WRAP participants undergo comprehensive medical, neuropsychological, and lifestyle evaluations, and they complete questionnaires every 2 years, as detailed in earlier literature.^26^ Eligibility required being CU at baseline, having both an amyloid PET scan and a FLAIR MRI, and having estimated onset ages for amyloid and vascular burdens (*n* = 240).

The Aim 4 replication involved 123 WADRC participants matching Aims 2 and 3 criteria (*n* = 123). Recruitment for the WADRC commenced in 2009, with participants typically attending annual follow-up visits. However, for those categorized as CU and <65 years old, assessments occur biennially. A flow chart detailing the participant inclusion criteria for each aim is shown in Supplementary Fig. 1.

For both WRAP and WADRC cohorts, participants’ written consent was obtained prior to study procedures according to the Declaration of Helsinki. This study was approved and conducted under the University of Wisconsin-Madison Institutional Review Board.

### Neuropsychological assessment protocols

Participants in each cohort completed a comprehensive neuropsychological test battery at each study visit. The WRAP test battery has been described elsewhere.^26^ WADRC uses the Uniform Data Set recommended by the National Alzheimer’s Coordinating Center^27^ and collects some additional measures in common with WRAP including the Rey Auditory Verbal Learning Test (AVLT).^28^ In this study, the primary cognitive outcome for both cohorts is a three-test modification of the Preclinical Alzheimer’s Cognitive Composite.^29^ In WRAP, the three contributing tests (PACC3) include the sum of AVLT Trials 1–5,^28^ Wechsler Memory Scale-Revised Logical Memory Delayed Memory score,^30^ and the Wechsler Adult Intelligence Scale-Revised Digit Symbol test score.^31^ In WADRC, it is derived using the sum of AVLT trials 1–5, WMS-R Logical Memory IIA in Uniform Data Set Version 2 or cross-walked Craft Story 21 in Uniform Data Set Version 3, depending on availability,^32,33^ and the Trail-Making Test Part B.^34^ The composites are derived by averaging standardized versions of the contributing tests as described previously.^35,36^

The following additional cognitive outcomes were included from WRAP as secondary outcomes for Aim 2 analyses. We analysed three domain-specific cognitive composites: executive function (EF), immediate memory, and delayed memory (the tests contributing to each of these composites are described in Clark *et al*.^37^ and Du *et al*.^38^).

### Cognitive status determination

Participant cognitive status in WRAP was established by a two-tiered monthly consensus conference process following each study visit as previously reported.^39^ Briefly, If neuropsychological assessments indicated potential abnormalities, a multidisciplinary team reviewed the data to diagnose cognitive states based on predefined criteria, distinguishing between Mild Cognitive Impairment (MCI)^40^ and dementia.^1^ Participants not meeting MCI or dementia criteria and without impairments from other conditions were classified as CU. The WADRC includes a similar multi-disciplinary consensus conference review of performance after each visit and uses the same diagnostic criteria for MCI and dementia.

### Neuroimaging data

All participants included in these analyses underwent MRI and amyloid PET imaging. MRI procedures, WMH quantification, and amyloid PET procedures are described below.

#### MRI assessment of white matter hyperintensities

Participants underwent MRI (Signa MR-750 or Premier using a 32-channel or 48-channel coil, respectively) including volumetric (3D) T1-weighted and T2 FLAIR acquistions at the University of Wisconsin-Madison Waisman Center Brain Imaging Lab. T1-weighted images were bias corrected, tissue class segmented and spatially normalized to MNI152 standard space (SPM12; www.fil.ion.ucl.ac.uk/spm). Detailed methods for amyloid PET and MRI acquisition, processing, quantification, and analysis were previously reported.^41^ MRI data were reviewed for image quality and exclusionary or actionable incidental findings by a board-certified neuroradiologist with >5 years clinical experience (Howard A. Rowley, Aaron Field, Laura Eisenmenger).

WMH lesion volume was segmented and summed using the automated lesion prediction algorithm from the Lesion Segmentation Tool version 3.0.0 for SPM12 in MATLAB (MATLAB v.R2018b; MathWorks) from T2-weighted FLAIR scans, and output as lesion probability maps. These maps were thresholded at a kappa of 0.5 (default lesion prediction algorithm recommendation) and the total volume of WMH (in millilitres, mL) was extracted without direct consideration of infarcts or lacunar lesions. For quality control, each lesion probability map was visually inspected (threshold at 0.5) and compared with the T2-FLAIR images.

The WMH raw volume was normalized to total intracranial volume (TICV: sum of grey matter, white matter and CSF volumes from SPM segmentation) to account for variability in brain size that may influence WMH measurements. It was then expressed as a percentage of TICV and multiplied by the average TICV to obtain the result in mm^3^ [Adjusted WMH = WMH/TICV*mean(TICV)]. In separate analyses of a larger, overlapping sample of *n* = 931, a two-class Gaussian mixture model was fitted to most recent adjusted WMH data using the Mclust package in R.^42^ We chose this approach to establish a binary cut-off for presence or absence of WMH because it allows for data-driven identification of a threshold and is a technique that is commonly used in biomarker research where biomarker outcomes typically represent bimodal distributions of overlapping values for people with and without the biomarker abnormality. The adjusted WMH volume corresponding to the intersection of the probability density functions of the two classes was used to classify participants into V− and V+ groups (adjusted WMH ≥2.06 mL indicates V+; Supplementary Fig. 2).

#### PET amyloid

Brain amyloid burden was assessed as a global average [11C]—Pittsburgh Compound B (PiB) PET. Detailed methods for amyloid PET acquisition, processing, quantification and analysis were implemented as previously reported.^41^ A multi-ROI DVR value derived from a dynamic scan of 0–70 min, taken across eight bilateral cortical regions of interest.^43^ For translatability, DVR values were also linearly translated to Centiloids using the equation: Equivalent Centiloids = 148.33×DVR−154.9621. In these analyses, PiB positivity (A+) was defined as a global 11C-PiB DVR ≥ 1.19 (∼21.6 Equivalent Centiloid).^44^. Longitudinal PET data were included in the analysis if people had at least one WMH value.

### Statistical analyses

Statistical analyses were conducted in R v4.0.2.^45^

#### Aim 1 analyses: characterizing WMH trajectories

In Aim 1 analyses, we extracted the estimated V+ onset ages and chronicities corresponding to ages at scans from the SILA-aligned data from the combined WRAP and WADRC cohorts (*n* = 877, all cognitive statuses included in Aim 1). Details on the SILA (https://github.com/Betthauser-Neuro-Lab/SILA-AD-Biomarker) method and validation in three amyloid PET samples are described in Betthauser *et al*.^21^ Briefly, the SILA algorithm applies discrete sampling to observed longitudinal data to establish the first-order relationship between the rate of biomarker change and the biomarker level. This curve is then smoothed and numerically integrated using Euler’s method to generate a nonparametric biomarker value versus time curve. The initial condition of time = 0 years at the biomarker threshold is then applied to the modelled curve. Individualized estimates of time from threshold are achieved by solving the value versus time curve for the observed value at a reference observation (last scan). Age of biomarker onset is calculated as age at reference observation minus time from threshold. For non-reference observations, time from threshold is calculated by subtracting the time difference between the observation and the reference observation (ageref—ageobs) from the time from threshold at the reference observation. Thus, time between observations within a person is preserved. Therefore, it would be easier to examine the temporal relationship between WMH and PiB amyloid using chronicity (age at scan—age of biomarker onset). The SILA-modelled WMH and PiB DVR curves are depicted in Supplementary Fig. 3A and B, respectively. To characterize WMH trajectories, we compared parameter estimates for mixed effects models using age or V chronicity as the time scale (up to a cubic polynomial for each) and interaction with V status (V+/V−). For each model, after examining model diagnostics of untransformed data, normalized WMH (adjusted WMH) volume was log transformed to reduce skewness. When an interaction with polynomial time was significant, we compared the simple slopes of V- and V+ WMH trajectories at specific overlapping time values using the ‘emmeans’ package.^46^

Of note, the chronicities in the V− group are not thought to represent actual time to V+ since only a small subset of that group will develop WMH’s in upcoming years; the negative values are included, however, as a means of modelling longitudinal measurement variability in people who are V− and in a way that allows direct comparison with V+ annualized change. See supplement for additional analyses comparing estimates of annual variability in WMH’s in the V− subset when using various operationalizations of time (e.g. age, time to last scan, chronicity).

#### Aim 2 analyses: temporal relationship of WMH and amyloid accumulation

Sample characteristics and the last cognitive status among those who were CU at their baseline cognitive assessment in WRAP were compared across A/V groups of interest (i.e. A−V−; A−V+; A + V−; A + V+) using tests appropriate for the distribution of the data. Tests included chi-square, fisher’s exact, analysis of variance (ANOVA) and Kruskal-Wallis. Significant omnibus tests (*P* < 0.05) were followed with pairwise comparisons. The onset ages of V and A were depicted in boxplots for the V+ or A+ subsets.

##### Aim 2a analyses: comparison of time operationalizations to understand the WMH trajectories

To investigate how amyloid burden relates to WMH trajectories in a CU population, especially in order to better understanding the temporal relationship between V and A, time at each WMH scan was operationalized in four ways to replicate various approaches used in the literature, including (i) age at which WMH was initially quantified, (ii) ∼V+ chronicity, (iii) years since baseline scan (covarying baseline age), and (iv) ∼A+ chronicity. For each time operationalization, a full model was examined that included linear and quadratic time terms and their interactions with PET A status at baseline WMH MRI to test whether time-related slopes differed between A+ and A− subsets (mixed effects models; random slopes and intercepts). Additional terms were included as follows in each ‘full model’ where the number corresponds to the time operationalization used.

Model 1: WMH = age*A status + age^2^* A status;

Model 2: WMH = Baseline WMH age + V chronicity*A status + V chronicity^2^ * A status;

Model 3: WMH = A status*baseline WMH age*time since first WMH scan;

Model 4: WMH = baseline WMH age + A status*A chronicity + A status * A chronicity^2^.

For each full model, after examining model diagnostics of untransformed data, normalized WMH (adjusted WMH) volume was log transformed to reduce skewness, and non-significant highest order interactions followed by quadratic time terms were removed sequentially; A status and time main effects were retained in each model. We depicted significant interactions for each time operationalization using simple slopes estimates. Data are plotted on the original scale and predicted values are back-transformed when they are depicted. When an interaction with quadratic time was significant, we compared the simple slopes of WMH trajectories at specific time values. We summarize final model results for the best fitting model(s) from Models 1, 3 and 4 using the corrected Akaike information criteria (AICc) and for Model 2 (if ΔAICc < 2 between best fitting and another model, results from both are described;^47^). Model 2 was left out of the AICc comparison because V chronicity’s mathematical relationship with raw WMH values in Model 2 results in very good model fit (by nature of the modelling that produced the WMH alignment) and the Model 2 AICc would therefore appear better regardless of whether A status contributed to variability in WMH trajectories. In addition, due to the large amount of variability accounted for by the time operationalization, there is less power to detect a significant A status*time interaction in this model. To address this Model 2 limitation, we conducted sensitivity analyses for Model 2 using V chronicity (linear and quadratic terms) to predict the WMH values separately in the A+ and A− subsamples, used the model output to estimate simple slopes (and 95% CI’s) at V chronicity values representing −10, −5, 0, 5 and 10 in the separate A+ and A− models, and depicted the overlap of these slope estimates in the A+ versus A− subsamples in a forest plot.

##### Aim 2b analyses: comparison of time operationalizations to understand the amyloid-PET trajectories

In Aim 2b, we performed parallel analyses of amyloid-PET trajectories (i.e. we followed the same steps as outlined in Aim 2a with longitudinal amyloid-PET as the outcome) to further examine the temporal relationship between A and V in CU participants. As in Aim 2a, the operationalization of time in Model 2 is chronicity corresponding to the outcome variable.

Model 1: Amyloid = age*V status + age^2^* V status;

Model 2: Amyloid = Baseline PiB PET age + A chronicity*V status + A chronicity^2^ * V status;

Model 3: Amyloid = V status*baseline PiB PET age*time since first PiB PET scan;

Model 4: Amyloid = baseline PiB PET age + V status*V chronicity + V status * V chronicity^2^.

#### Aim 3 analyses: examine how cognitive trajectories are modified by A and V status

After clarifying the temporal relationship between A and V in CU participants, we interrogated how A and V contribute to longitudinal cognitive decline. We investigated four cognitive trajectory (PACC3 global cognition (primary), EF, immediate memory, and delayed memory) differences in WRAP participants, initially CU, across four groups based on their last known status of PiB and WMH (A−/V−, A−/V+, A+/V−, A+/V+). Cognitive outcomes were analysed using mixed-effects models with random intercepts, correlated age-related slopes, and linear (and removed non-significant quadratic) age terms (centrerd on 60). The primary predictors were the A/V groupings and their interactions with age, using A−/V− as the reference. Covariates included sex, Wide Range Achievement Test-III Reading standard score (Wilkinson, 1993) (a proxy for educational quality), and practice effects (the number of prior exposures to the battery). The most parsimonious models excluded non-significant interactions and quadratic terms. Significant interactions prompted pairwise comparisons using Tukey-adjusted tests and simple slope estimations at ages 60, 70, and 80, with corresponding confidence intervals. The Kenward–Roger approximation was applied to ascertain the degrees of freedom.

Since we have previously shown that baseline A+ chronicity explains more variability in cognitive trajectories than binary A+ status,^23^ further sensitivity analyses incorporated A+ and V+ chronicity at last visit using continuous interactions, replacing the A/V group*age interaction. Non-significant higher order interactions were removed sequentially, and these models were assessed against the primary analysis model through AICc and likelihood ratio tests. When the three-way interaction was significant, we plotted the predicted cognition for the median of the negative and positive ranges of each biomarker.

#### Aim 4 analyses: replication in an independent cohort

To validate the temporal relationship between A and V and how A and V contribute to cognitive decline, we performed a replication analysis on WADRC participants (*n* = 123) who met the same inclusion criteria, examining how amyloid burden relates to WMH trajectories and whether PACC3 age-related trajectories differed by A/V± groups (at the last scan) in WADRC participants. The differences between WRAP and WADRC in sample characteristics, cognitive status, and time between biomarker onset ages (or onset of one and last negative) were examined using tests appropriate for the distributions of the variables. We repeated the analyses described in Aims 2 and 3 using this smaller sample. Simple slope estimates were extracted from both cohorts if the interaction was significant in either to compare the effects between the main study and the replication study.

## Results

### Aim 1: characterization of WMH trajectories

**Figure 1:**
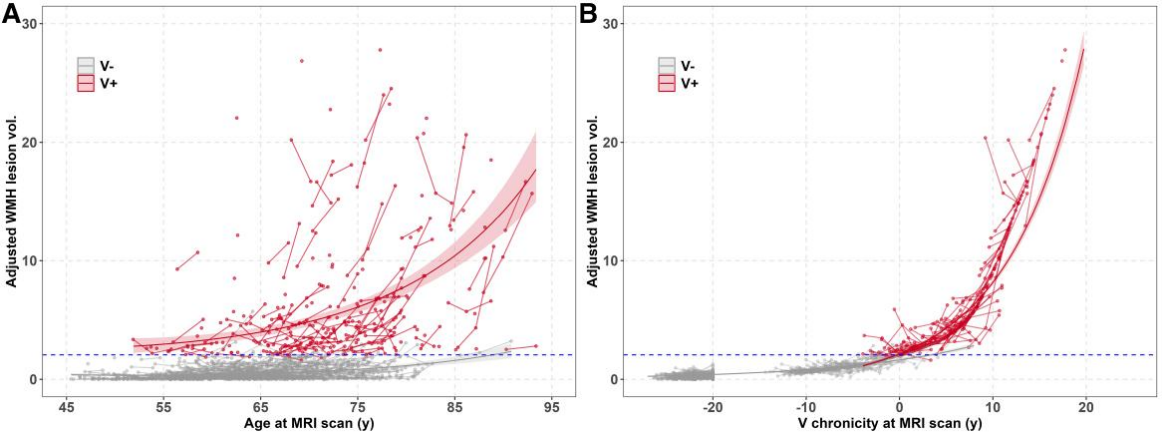
**The spaghetti plot of Aim 1 WMH lesion volume versus age and WMH versus SILA-aligned V chronicity at time of each scan, by V± group (*n* = 877).** (**A**) WMH versus age, by V± status. (**B**) WMH versus V chronicity at time of each scan (calculated as age at scan minus V+ onset age), by V± status. The plot overlays the predicted WMH values from the LME models that used either age or V chronicity as the time scale **A**. V status*age *beta* CI = 0.01–0.02, *P* = 0.001; **B**. V status*chronicity *beta* CI = 0.05–0.06, *P* < 0.001). *P*-values for fixed effects were calculated based on the asymptotic normal distribution properties of the estimates, and statistical significance was assessed using Wald tests. Please see Supplementary Table 2 for the model output, Supplementary Fig. 3A for the SILA-modeled curves described in Betthauser *et al.*, and Supplementary Fig. 4 for more information in V− subset. WMH/V, white matter hyperintensities; SILA, sampled iterative local approximation; LME, linear mixed effect model.

Figure 1A shows the longitudinal WMH spaghetti plot versus age at scan for the combined WRAP and WADRC samples used in SILA modelling (*n* = 877; 566 (64.5%) female; 87.8% CU; 6.3% MCI; 3.9% dementia; 1.8% impaired other; 0.2% no diagnosis; for additional Aim 1 sample details, see Supplementary Table 1; the SILA-modelled WMH curves are presented in Supplementary Fig. 3A). The WMH spaghetti plots versus V chronicity are shown in Fig. 1B. In separate mixed effects models and for both age and V chronicity time operationalizations, quadratic time was retained in the model along with V status*time (but not V status*time^2^; see model output in Supplementary Table 2). The V− and V+ prediction lines based on linear mixed effect model models are superimposed on the individual observations in Fig. 1 for each time operationalization (thick lines). Corresponding simple slopes analyses using the model outputs reveal that the slope (i.e. estimated annual change) for V− is fairly consistent whether using age or V chronicity. For example, simple slope(se) estimates at ages 60 and 75 in the V− group were 0.010 (0.002) and 0.028 (0.003), respectively; similarly, simple slopes were 0.020 (0.003) and 0.037 (0.001), respectively, for chronicities below −20 years (i.e. below the lowest modelled range) and −5 (i.e. a chronicity between the lower modelled range and the V+ threshold). Sensitivity analyses in the V− subset are shown in Supplementary Fig. 4, using three time operationalizatons to characterize estimates of annual change. The consistency across time estimates supports the validity of using chronicities to model V− and V+ changes together. In addition, the beta estimate for the V status*time interaction was much larger in the V+ group when using chronicity instead of age (V status*age *beta CI* = 0.01–0.02; V status*chronicity *beta CI* = 0.05–0.06; Supplementary Table 2), indicating that WMH volumes accumulate at a faster annual rate than predicted when using age as the time operationalization.

### Aim 2: temporal relationships of WMH and amyloid accumulation in a preclinical sample

The study sample for Aims 2 and 3 included 240 WRAP participants who were CU at their baseline cognitive assessment, had completed at least one cognitive assessment, and had at least one WMH and PiB DVR with corresponding estimated onset ages at the time of data export. Overall, the mean (SD) age at the last MRI was 69.2 (6.9) years and 68.7 (6.7) years at PiB PET. One hundred and sixty-five (68.8%) participants were female, 94 (40.3%) were APOE e4 carriers and 211 (92.6%) were cognitively unimpaired at last cognitive status (Table 1). Table 1 also compared the characteristics of the four A/V groups used in Aim 2 analyses to further characterize the sample (A−V−, A−V+, A + V− and A + V+). In the A− subset, 37(21.5%) were V+ compared with 16(23.5%) V+ in the A+ group. Similarly, in the V− subset, 52(27.8%) were A+ compared with 16(30.2)% A+ in the V+ group (χ^2^ test of association between A and V status *P* = 0.86; Supplementary Table 3). In general, while the chi-square showed A status and V status were not associated, A+ (28.3%) was more common than V+ (22.1%). There were no group differences in race, WRAT3 reading, or education (*P* > 0.05). More women were V+ and *APOE*4 was more common among A+.

**Table 1:** Aims 2 and 3 sample characteristics by A/V ± groups

Variables	Overall	A−/V−	A−/V+	A+/V−	A+/V+	*P*-value^a^
	n = 240	135(56.2%)	37(15.4%)	52(21.7%)	16(6.7%)	
Age at baseline WMH, mean (SD)	67.16 (6.78)	64.97 (7.12)	71.22 (4.53)	68.66 (5.53)	71.28 (3.92)	<0.001
Proportion A+ at WMH baseline, n (%)	61 (25.4)	0 (0.0)	0 (0.0)	46 (88.5)	15 (93.8)	<0.001
Age at last WMH, mean (SD)	69.17 (6.89)	66.82 (7.20)	73.30 (4.52)	70.81 (5.29)	74.19 (4.54)	<0.001
Years of WMH follow-up, mean (SD)	3.14 (1.08)	3.11 (1.03)	2.95 (0.86)	3.18 (1.18)	3.58 (1.44)	0.392
Age at last PiB, mean (SD)	68.72 (6.72)	66.47 (7.03)	72.96 (4.82)	69.99 (4.73)	73.82 (4.74)	<0.001
PiB Last Age—WMH Last Age, mean (SD)	−0.45 (1.59)	−0.35 (1.64)	−0.34 (1.26)	−0.82 (1.84)	−0.37 (0.70)	0.319
Years of PiB follow-up, mean (sd)	7.51 (2.89)	7.49 (3.03)	7.66 (2.58)	7.52 (2.66)	7.38 (3.46)	0.992
Female, n (%)	165 (68.8)	90 (66.7)	31 (83.8)	30 (57.7)	14 (87.5)	0.021
Family history, n (%)	170 (71.1)	94 (70.1)	20 (54.1)	42 (80.8)	14 (87.5)	0.021
White/Caucasian, n (%)	226 (94.6)	125 (93.3)	37 (100.0)	50 (96.2)	14 (87.5)	0.225
College degree, n (%)	166 (69.5)	95 (70.9)	22 (59.5)	38 (73.1)	11 (68.8)	0.532
WRAT3 reading score, mean(sd)	106.85 (8.98)	106.65 (9.30)	107.11 (8.27)	105.92 (9.18)	111.00 (6.36)	0.255
*APOE* e4 count, n (%)						<0.001
0 alleles	137 (59.8)	84 (67.2)	29 (78.4)	17 (33.3)	7 (43.8)	
1 allele	77 (33.6)	37 (29.6)	8 (21.6)	25 (49.0)	7 (43.8)	
2 alleles	15 (6.6)	4 (3.2)	0 (0.0)	9 (17.6)	2 (12.5)	
Last cognitive status, n (%)						<0.001
CU	211 (92.6)	126 (99.2)	33 (94.3)	41 (82.0)	11 (68.8)	
MCI	13 (5.7)	1 (0.8)	2 (5.7)	8 (16.0)	2 (12.5)	
Dementia	4 (1.8)	0 (0.0)	0 (0.0)	1 (2.0)	3 (18.8)	

Note: Global PiB DVR > 1.19 = A+. Estimates proportion A+ at WMH baseline scan use SILA modelling as described in Betthauser *et al*., 2022. TIV-adjusted WMH >2.06 = V+. The group A+−/V+− were created using the last WMH scan.

V; WMH, white matter hyperintensities; A, amyloid PET; PiB, Pittsburgh compound-B; WRAT3, Wide Range Achievement Test-III Reading Recognition subtest; CU, cognitive unimpaired; MCI, mild cognitive impaired; A, amyloid; V, cerebrovascular.

^a^Statistical tests: χ^2^ or Fisher’s exact for categorical; analysis of variance (ANOVA) for continuous where M (SD) or lsmean (SE) reported; Kruskal–Wallis for continuous where median [Q1–Q3] reported and Likert-scale items.

Estimated onset ages are shown in Supplementary Fig. 5 for those who are V+ (top panel; estimated V+ onset ages (EVOA) range = 51.8–78.7) and those who are A+ (middle panel, estimated A+ onset ages (EAOA) range = 40.8–76.2). The bottom panel depicts age at last MRI/PET and ranged from 46.4 to 81.0 for those who were A−/V− as of last scan. In the subset that was A+ and V+ (*n* = 16(13.2%)), the median[Q1–Q3] years from EAOA to EVOA was −4.64 [−11.58, −0.27], indicating that A+ tended to precede V+ in this subset.

#### Aim 2a: investigating A+ versus A− differences in V trajectories by each of four-time operationalizations

**Figure 2:**
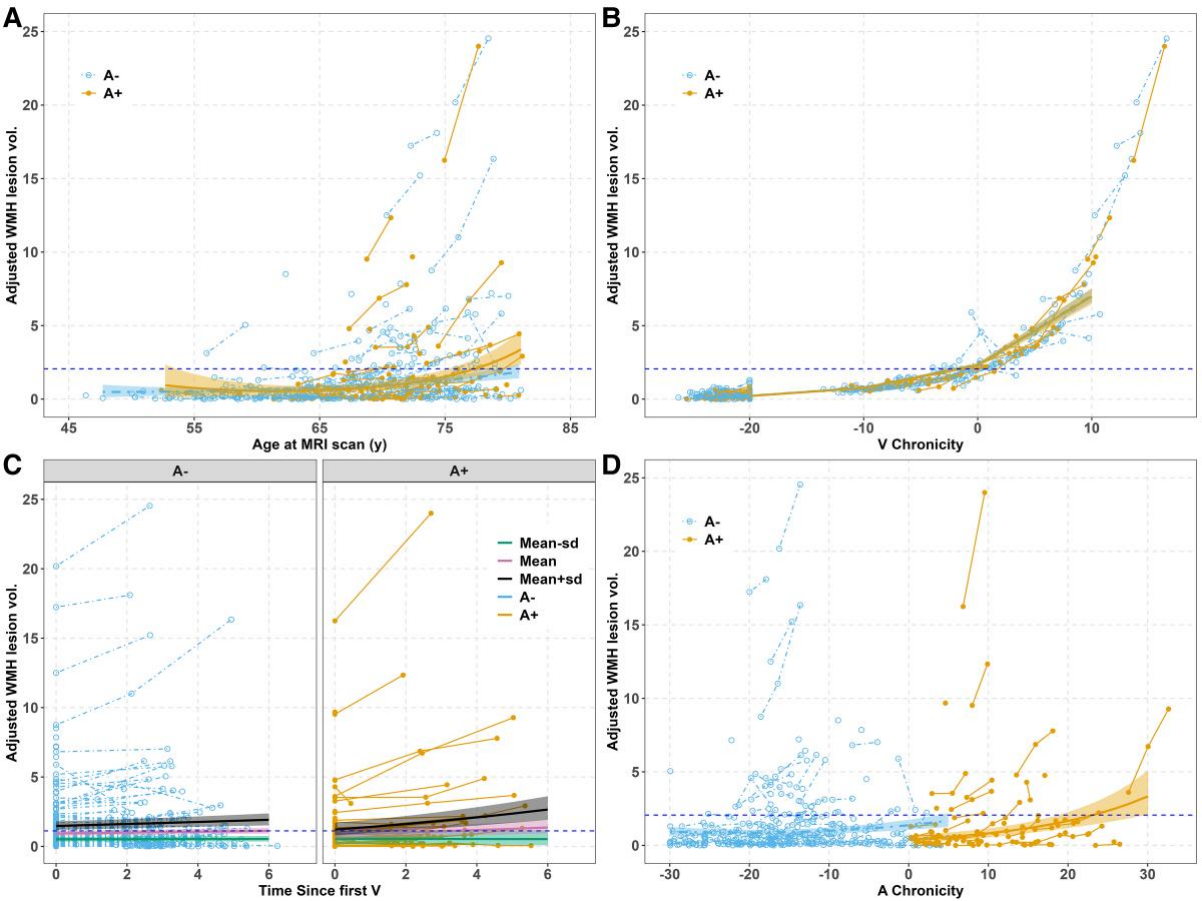
**Aim 2a WMH spaghetti plots and predicted slopes from mixed effects for four time operationalizations (WRAP; *n* = 240).** (**A**) WMH versus age, by A± status. Trajectories show WMH typically begins later in life. Simple slopes analyses indicate that A+ and A− WMH slopes differ significantly at ∼ age 70 and after (A status*age *beta* CI = −0.03 to 0.02, *P* = 0.861; A status*age^2^  *beta* CI = 0.00–0.00, *P* = 0.011). (**B**) WMH lesion volume versus SILA-aligned V+ chronicity (labelled ‘Years V+’) at time of each scan (calculated as age at scan minus V+ onset age). No significant difference between A+ and A− WMH trajectories. Note: To include those who are V− in the visualization, ‘Years V+’ was truncated to −20 for last scan of those who are V−. (**C**) Simple slopes plot of significant baseline WMH age*A status*years since baseline WMH interaction (A status* baseline age* time since first V *beta* CI = 0.00–0.01, *P* = 0.015). Baseline WMH ages depicted are: 60.5 (mean−1 SD), 67.1 (mean) and 73.6 (mean + 1 SD). Patterns suggest WMH increases faster in A+ versus A− in those who are older but not in those who are younger. (**D**) WMH versus A+ chronicity at WMH scans. WMH slopes (estimated annual change) differ between those who are PET A− and those who are PET A+ (A+ status *beta* CI = −0.81 to (−0.27), *P* < 0.001). Please see Supplementary Table 4 for the model outputs of the final models corresponding to the WMH trajectories and the four time operationalizations. WMH, white matter hyperintensities; TICV, total intracranial volume; PiB, Pittsburgh compound-B; SILA, sampled iterative local approximation.

Model outputs for the final models corresponding to the WMH trajectories and the four-time operationalizations are shown in Supplementary Table 4 and prediction lines based on these models are depicted on spaghetti plots of observed trajectories in Figs 2A–D. We focus here on results from Model 3 (time = Time since baseline; the best-fitting model of Models 1, 3 and 4 with *ΔAICc*≥−10.6) and Model 2. In Model 3, the three-way baseline WMH age*time since baseline scan*PET A status interaction was significant (Supplementary Table 4). The simple slope estimates of average WMH annual change are depicted in Fig. 2C for baseline ages at the mean baseline age (=67.06), the mean ± 1 SD (baseline ages = 60.52 and 73.6), and by A ± status. The A−/A+ simple slopes estimates differ only at older ages with faster estimated average annual WMH accumulation in A+’s (e.g. slope estimates at time since baseline 10 years are 0.056 for A− and 0.093 for A+; the 95% CI for the A- minus A+ difference in slopes at this age is 0.012–0.061).

While linear and quadratic V Chronicity terms were significant in Model 2, PET A status*V chronicity terms were not significant (i.e. did not show support for faster accumulation in those that were A+ compared with A−). Sensitivity analysis for Model 2 showed that the WMH slopes at V chronicities of −10, −5, 0, 5, and 10 were similar in both the A+ and A− subset models (Supplementary Table 5 and Supplementary Fig. 6A). For example, the estimated simple slopes from the separate A− and A+ models were as follows for A+/A− (at −5 or 5 years of V chronicity): 0.066/0.062 (−5 years of V chronicity); 0.094/0.086 at 5 years V chronicity.

#### Aim 2b: amyloid PET trajectories by each of four-time operationalizations

**Figure 3:**
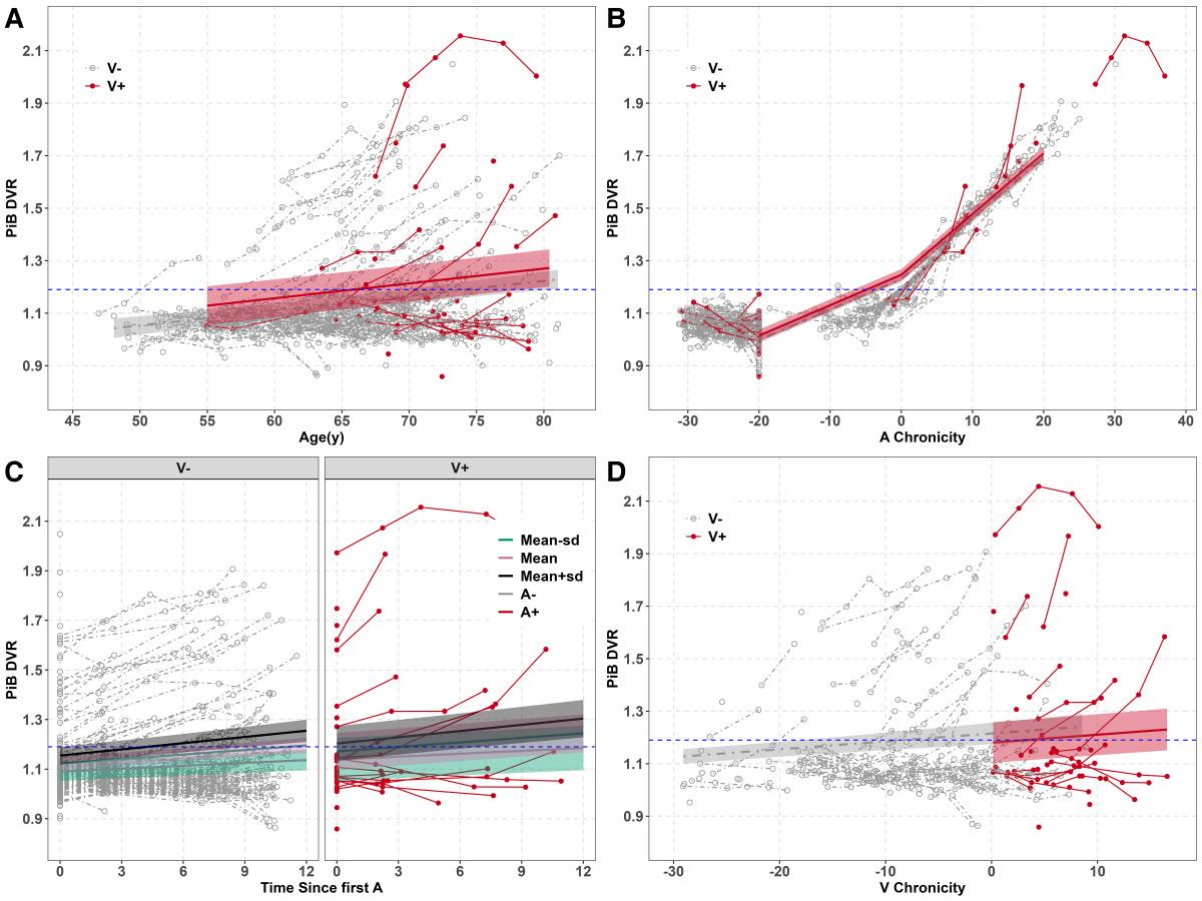
**Aim 2b PiB spaghetti plots and predicted slopes from mixed effects for four time operationalizations (WRAP; n = 240).** (**A**) PiB DVR versus age, by V± status (V+ status *beta* CI = −0.03 to 0.12, *P* = 0.220). (**B**) PiB versus SILA-aligned A+ chronicity at time of each scan (calculated as age at scan minus A+ onset age). The predicted lines are based on LME model and are not the same as the SILA-modeled curves described in Betthauser *et al.* 2022; for these curves, please see Supplementary Fig. 3B. No significant difference between V+ and V− PiB trajectories. Note: To include those who are A− in the visualization, ‘PiB chronicity’ was truncated to −20 for last scan of those who are A−. (**C**) Simple slopes plot of significant baseline PiB age*V status*years since baseline PiB interaction (baseline age* time since first A *beta* CI = 0.0001–0.0006, *P* = 0.015). Baseline PiB ages depicted are: 55.4 (mean−1 SD), 62.1 (mean), and 68.8 (mean + 1 SD). (**D**) PiB DVR versus V+ chronicity at PiB PET scans. PiB slopes (estimated annual change) do not differ between those who are V− and those who are V+(V+ status *beta* CI = −0.12 to 0.05, *P* = 0.435). Please see Supplementary Table 6 for the model outputs of the final models corresponding to global PiB DVR trajectories and the four time operationalizations. WMH, white matter hyperintensities; TICV, total intracranial volume; PiB, Pittsburgh compound-B; PET, positron emission tomography; DVR, distribution volume ratio; SILA, sampled iterative local approximation.

Model outputs for the final models corresponding to global PiB DVR trajectories and the four-time operationalizations are shown in Supplementary Table 6 and prediction lines based on these models are depicted in Figs 3A–D. The quadratic time term was significant and retained only in Model 2. Model 3 (Time = Time since baseline) had *ΔAICc*’s < 2 with Model 1 (Time = Age), suggesting similar model fit. In Model 3, the three-way V status*baseline PiB age*time since baseline PiB was not significant and after sequential removal of that and other non-significant interactions, the model showed only an association between baseline PiB PET age and PiB PET annual accumulation; there were no differences between those who were V+ at baseline PiB PET versus V− at that time (Supplementary Table 6 and Fig. 3C). Model 1 (time operationalization = age at each PiB PET) also did not identify a significant V status*age interaction, suggesting that age-related PiB trajectories were not different on average for those who are V+ at baseline PiB PET scan versus V− at that time (Supplementary Table 6 and Fig. 3A). Model 4 (*ΔAICc* = 27.5 compared with Model 3), after accounting for baseline PiB PET age, PiB trajectories did not differ by V status at baseline PiB PET when V+ chronicity was also taken into account in the operationalization of time in model (Supplementary Table 6 and Fig. 3D).

Similar to Model 2 with the WMH outcome, the linear and quadratic A Chronicity terms were significant in Model 2 but no significant A Chronicity* V status or significant main V effect were retained in the model (Supplementary Table 6, Fig. 3B). Sensitivity analysis for Model 2 showed that the slopes of A chronicity at −10, −5, 0, 5 and 10 were similar in both the V+ and V− subset models (Supplementary Table 7 and Supplementary Fig. 6C). For example, the estimated simple slopes from the separate V− and V+ models were as follows for V+/V− (at −5 or 5 years of A chronicity): 0.015/0.014 at −5 years of A chronicity, and 0.021/0.020 at 5 years.

### Aim 3: cognitive trajectories by age and biomarker information

**Figure 4:**
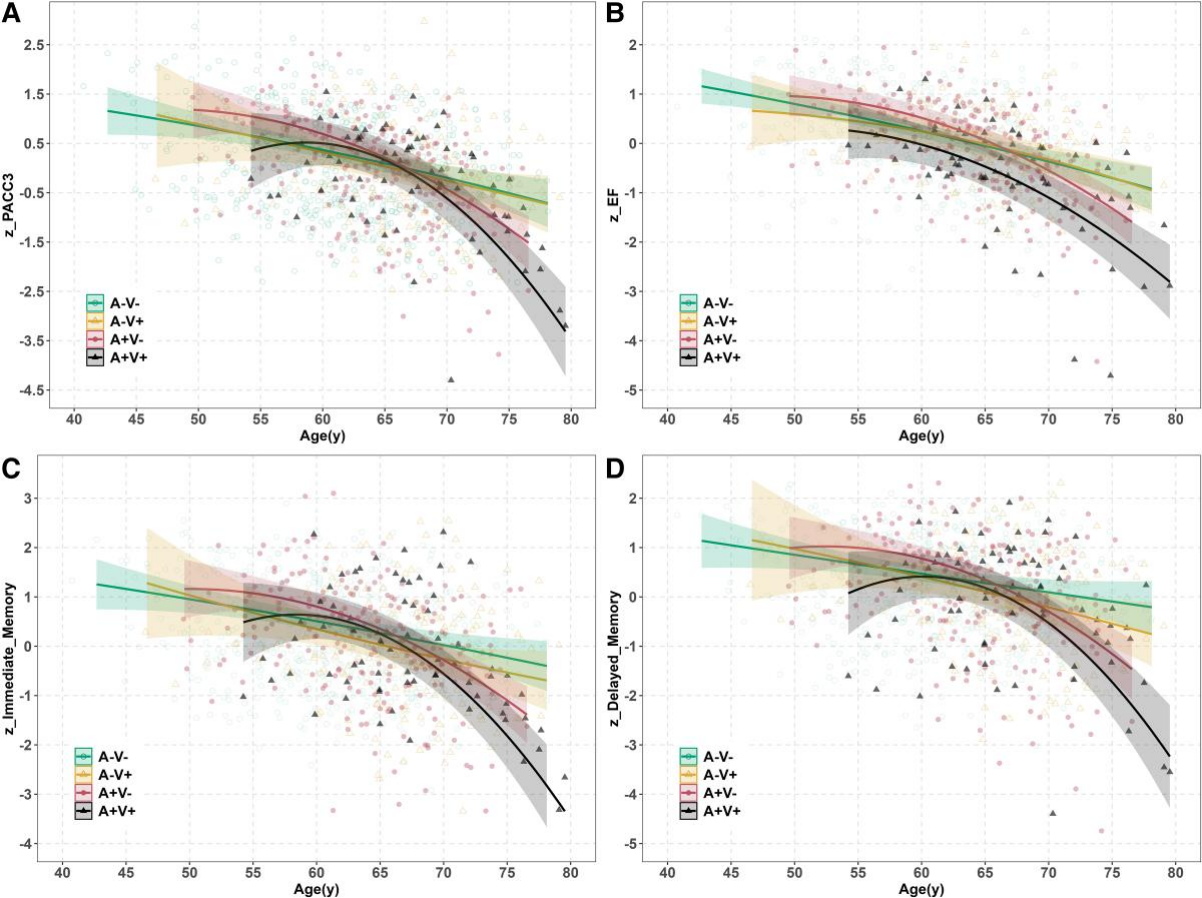
**Aim 3 observed cognitive composite *z*-scores (dots) and simple slopes for A/V groups from mixed models.** (Model was cognitive composite *z*-scores ∼ c60_age * A/V groups + linear and quadratic c60_age + Sex + WRAT3+ education + number of prior exposures + random person-level intercepts and age slopes; c60_age indicates age centred at 60.) (**A**) PACC3 (A-V+*age^2^  *beta* CI = −0.00–0.00, *P* = 0.804; A + V−*age^2^  *beta* CI = −0.01 to −0.00, *P* = 0.027; A + V+*age^2^  *beta* CI = −0.01 to −0.00, *P* < 0.001); (**B**): EF (A−V+*age^2^  *beta* CI = −0.00–0.00, *P* = 0.424; A + V−*age^2^  *beta* CI = −0.00 to −0.00, *P* = 0.002; A + V+*age^2^  *beta* CI = −0.01 to −0.00, *P* = 0.019); (**C**) Immediate Memory (A−V+*age^2^  *beta* CI = −0.00 to 0.00, *P* = 0.681; A+V−*age^2^  *beta* CI = −0.01 to −0.00, *P* = 0.009; A+V+*age^2^  *beta* CI = −0.01 to −0.00, *P* < 0.001); (**D**) Delayed Memory (A−V+*age^2^  *beta* CI = −0.00 to 0.00, *P* = 0.846; A+V−*age^2^  *beta* CI = −0.01 to −0.00, *P* = 0.003; A+V+*age^2^  *beta* CI = −0.01 to −0.01, *P* < 0.001). Please see Supplementary Table 8 for final model output of each cognitive outcome. A, amyloid PET; V, white matter hyperintensities; PACC3, Preclinical Alzheimer’s Cognitive Composite (comprised of averaged *z*-scores for three tests: Auditory Verbal Learning Test (AVLT) Total Learning, Logical Memory Delayed Memory, and Digit Symbol Substitution); EF, executive function (comprised of averaged *z*-scores for three tests: Stroop Color-Word, Trail Making Test Part B and Digit Symbol Substitution).

**Figure 5:**
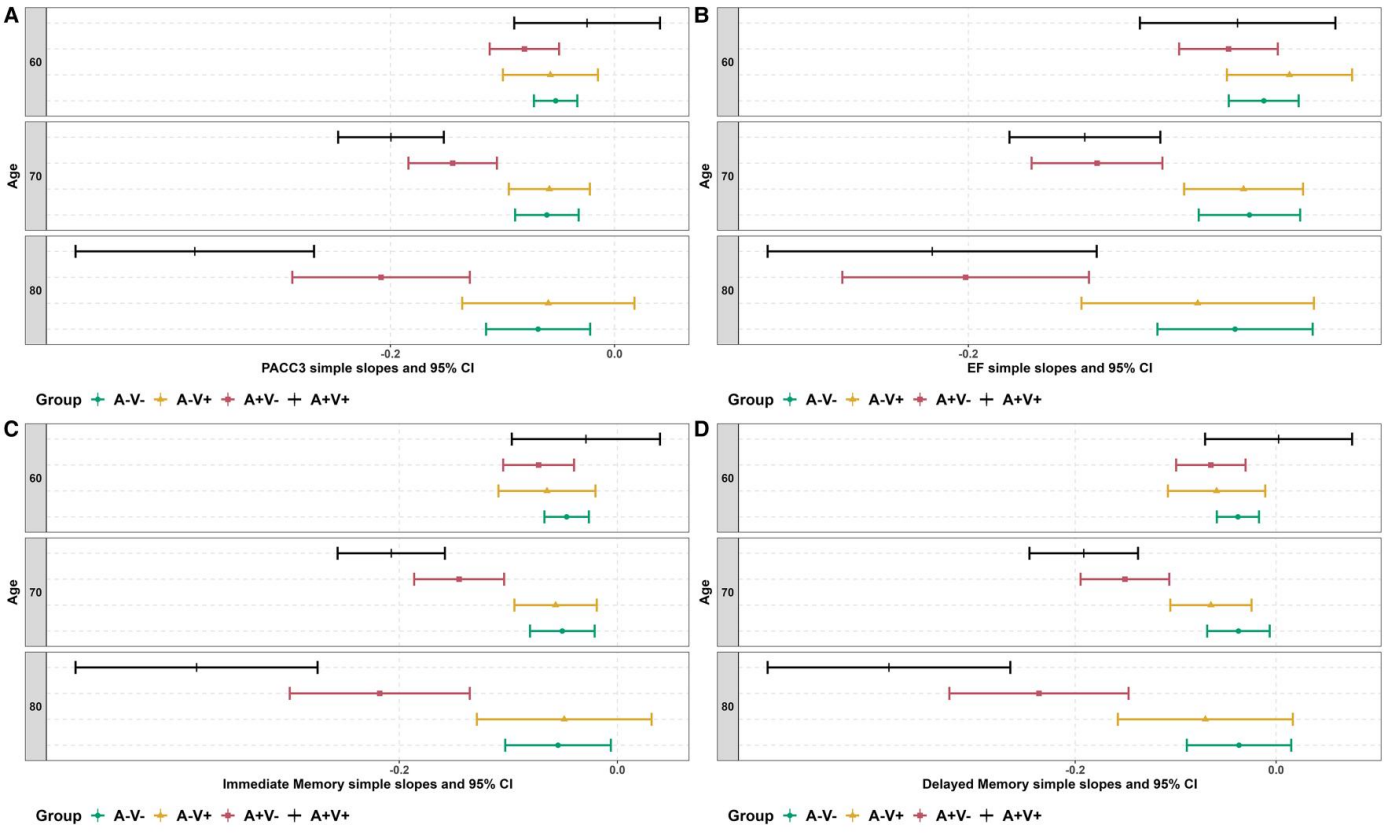
**Simple slopes estimating per-year change in cognition as a function of age and A/V group.** (**A**) PACC3, (**B**) EF, (**C**) immediate memory, (**D**) delayed memory. Estimated PACC3 simple slopes (CIs) at ages 60, 70 and 80 for each group were: A−V−; −0.0525 (−0.0719, −0.0332), −0.0604 (−0.0887, −0.032) and −0.0682 (−0.1146, −0.0217); A−V+; −0.0572 (−0.0996, −0.0148), −0.0581 (−0.0943, −0.022) and −0.0591 (−0.1359, 0.0178), A+V−; −0.0804 (−0.1114, −0.0495), −0.1444 (−0.1839, −0.1049) and −0.2083 (−0.2875, −0.1291) and A+V+; −0.0245 (−0.0895, 0.0406), −0.1995 (−0.2466, −0.1523) and −0.3745 (−0.4809, −0.2681). A, amyloid PET; V, white matter hyperintensities; PACC3, Preclinical Alzheimer’s Cognitive Composite (comprised of averaged *z*-scores for three tests: Auditory Verbal Learning Test (AVLT) Total Learning, Logical Memory Delayed Memory, and Digit Symbol Substitution); EF, executive function (comprised of averaged z-scores for 3 tests: Stroop Color-Word, Trail Making Test Part B and Digit Symbol Substitution).

The final model for PACC3, the primary cognitive outcome, showed that A/V groups (at last scan) interacted with both linear and quadratic age (see Supplementary Table 8 for model output and Fig. 4A for A/V group-specific regression lines superimposed on a scatter plot of the data). People in the A+/V+ group had the highest rates of decline at older ages, followed by A+/V− compared with people in the A−/V− group (Fig. 5).

Patterns were similar for most of the secondary cognitive outcomes. Specifically, A/V group interacted significantly with age for the three domain-specific cognitive composites EF (Fig. 4B), Immediate memory (Fig. 4C) and Delayed memory (Fig. 4D) (see also Supplementary Table 8 for model output for each outcome). The simple slopes from significant interactions, as depicted in Fig. 5 for each cognitive outcome, reveal that people in the A+/V+ group experienced markedly higher rates of decline, especially after age 70, for all outcomes.

In the sensitivity analysis replacing the A/V group variable with the two continuous A and V chronicity variables, the final model for PACC3 included significant three-way interactions with both linear and quadratic age. The model incorporating both A+ chronicity and V+ chronicity had a similar AICc value compared with the primary model depicted in Fig. 4A (i.e. depicting simple age-related slopes by A/V groups) (Supplementary Table 9; *ΔAICc* = 0.2). Simple slopes analyses categorized by A/V chronicity are depicted in Supplementary Fig. 7, with four distinct groups (A/V chronicity = −10/10 separately). The patterns are similar to the simple slopes observed in the A/V group*time model, with the group where both A and V chronicity are set to 10 showing the most pronounced decline, especially at ages.

### Aim 4: replication

Our replication sample included 123 WADRC participants who were CU at their baseline cognitive assessment, had completed at least one T1-weighted MRI and T2-weighted FLAIR scan as well as at least one amyloid PET scan, and had completed at least one cognitive assessment. The mean (SD) last MRI age was 68.4 (8.4) years. 67.80 (6.99) years at PiB PET. Eighty-eight (71.5%) participants were female, 48 (40.7%) were APOE e4 carriers and 116 were CU (94.3%) at last cognitive status. Supplementary Table 1 showed the characteristics by WRAP and WADRC data. There were no significant differences in descriptive characteristics, except family history and race (*P* < 0.05). Similar to the WRAP sample, a chi-square test showed that there was no significant association between PiB positivity and WMH positivity in WADRC sample (Supplementary Table 3).

In the replication of Aim 2a analysis, model outputs for the final models corresponding to the four-time operationalizations are shown in Supplementary Table 10 and depicted in Supplementary Fig. 8A–D. Model 3 (Time = Time since baseline) had *ΔAICc’s* < 2 with Model 1 (Time = Age), suggesting similar model fit. In Model 3, the association between baseline WMH age and WMH annual accumulation did not differ between those who were PET A+ at baseline WMH versus PET A− at that time (Supplementary Table 10 and Supplementary Fig. 8C). Model 1 (time operationalization = age) WADRC results differed from WRAP results in that they did not show age-related WMH trajectory differences on average for those who are PET A+ at baseline MRI scan versus PET A− at that time (Supplementary Table 10 and Supplementary Fig. 8A). While Model 4 was not as good a fit as either Models 1 or 3 (*ΔAICc* ≥ 15.7), it is interesting to note that ADRC results differ from corresponding WRAP results. Specifically, the estimated change in WMH was higher among A+’s.

Similarly, no significant V Chronicity* PET A status was retained in the model and the main PET A effect was not significant (Supplementary Table 10 and Supplementary Fig. 8B). Similar to the results in WRAP sample, sensitivity analysis for Model 2 showed that the slopes of V chronicity at −10, −5, 0, 5 and 10 were similar in both the A+ and A− subset models (Supplementary Table 5 and Supplementary Fig. 6B).

In the replication of Aim 3 analysis, the final model for PACC3 included a linear and quadratic age term and showed that there was a significant A/V status*age interaction (Supplementary Table 11). Simple slopes analyses again showed PACC3 decline was fastest on average in the A+/V+ group (Supplementary Fig. 9).

## Discussion

In this work, we examined the temporal relationship between cerebrovascular disease (or V) as evidenced by abnormal WMH and amyloid burden (or A), which is typically the first indicator of Alzheimer’s disease and may occur one to three decades prior to dementia. We first derived age of biomarker onset and chronicity estimates for WMH and compared this operationalization of time to other time operationalizations. We then examined their impact on cognitive trajectories in an initially CU sample of late-middle-aged adults enriched for risk of Alzheimer’s disease. A main conceptual advance from this work is that, like amyloid,^21^ V onset age and chronicity can be estimated from WMH lesions of presumed vascular origin. We found that V and A, though both age-related processes, are largely independent from one another such that the presence or trajectory of one did not predict the presence or trajectory of the other, particularly in younger ages; that when both are present, A+ typically occurs first by 4.5 years on average, and also that when both are present, cognitive decline is greater and is dependent on the chronicity of the biomarkers with amyloid being the most salient of the two predictors. These findings provide crucial insight on the temporal relationships between A and V in the brain and their joint effect on cognitive decline.

In longitudinal studies, chronological age itself may be an intuitive indicator of progression of disease or lack thereof, and here such general age-related effects were noted. Moreover, we found that WMH accumulation was slightly faster in A+ compared with A− individuals at older ages, suggesting potential unidentified age-related mediators in this relationship that may be related to manifestation or progression of disease at older age among the cohorts studied. However, age itself is imperfect when examining the temporal relationships between these diseases because A+ onset and V+ onset can each occur across the range of middle age to older age. Indeed, Betthauser *et al*.^21^ observed that A+ onset can vary widely and occurred variably across a 50-year span from age 40 to 90 (see also^23^). Analogously, in this study we observed that V+ onset age varied from age 52 to 79. Similar limitations are evident when time is operationalized relative to an age-at-enrolment referent such as the age range allowable by protocol or the observed age range, or even the duration of time on the study. These factors commonly vary within longitudinal cohort studies and certainly do in each of the two cohorts presented here.

In contrast, when time was operationalized as chronicity (time with disease), the temporal relationships between A and V became quite clear. In parallel models, neither did WMH lesion volume growth trajectories differ by A+ status, nor did amyloid trajectories differ by V+ status. Furthermore, although in a proportion of cases V and A may coexist in the same brain at some point in time (and more likely with advanced age), their onset ages actually differ significantly within individuals, with amyloid generally but not always occurring first (median of 4 years). Our observation of a lack of a difference in WMH lesion volume trajectories by A+ status and vice versa suggests that these processes occur independently. However, the presence of both A+ and V+ was associated with the sharpest cognitive decline in CU individuals and driven primarily by additive amyloid burden.

Our findings extend previous studies exploring the association between A and V, including recent work from the Dominantly Inherited Alzheimer Network and the Alzheimer’s Disease Neuroimaging Initiative projects, which linked WMH with Alzheimer’s disease biomarkers.^12^ However, our study supports the notion that the coexistence of these pathologies does not imply a causal relationship between them. This contrasts with recent studies.^48-52^ suggesting a mechanistic link between white matter changes and Alzheimer’s disease pathologic features. While WMH is considered an early feature of Alzheimer’s disease in studies of autosomal dominant Alzheimer’s disease mutation carriers^13^ our findings align with those from the Mayo Clinic which showed that, despite the coexistence of WMH and amyloid or tau in similar brain regions, there was no temporal relationship in their onsets or progression, suggesting independent mechanisms.^17^

Further studies have also refuted the hypothesis of a mechanistic association between A and V. For example, a recent study found that vascular risk was associated with white matter lesion load, but not amyloid-β or tau,^16^ while another cross-sectional imaging study found that diffusion metrics were associated with age and WMH, but not with amyloid or tau.^53^ Additionally, a meta-analysis indicated that amyloid deposition and V/WMH are independent, additive processes contributing to cognitive decline.^15^ A twin study also found no genetic or environmental link between amyloid burden and V, as measured by WMH volume and mean diffusivity.^54^

Notably, the presence of both A+ and V+ was associated with the sharpest cognitive decline in CU individuals, driven primarily by additive amyloid burden. Previous studies have similarly found that vascular risk and Aβ pathology have additive, rather than synergistic, effects on cognitive decline.^55,56^ Several studies also suggest that vascular risk acts independently of Alzheimer’s disease biomarkers in contributing to cognitive decline.^57,58^ Therefore, the presence of both A and V is harmful, making them critical targets for intervention aimed at preventing or slowing clinical progression. Understanding the distinction between these disease processes is essential for the development of future targeted therapies.

These findings were validated in an independent cohort (WADRC). In both the WRAP and WADRC, when time operationalized as V+ chronicity, the growth of WMH lesion volume was similar for both A+ and A− groups. Additionally, the growth rates at different chronicity levels were alike for both groups. Similarly, the presence of both A and V was associated with fastest cognitive decline in the replication sample. Minor observed differences in the outcomes of WMH trajectories when we use age or A chronicity as timescale across the cohorts could be attributed to variations in cohort study design, including regional and demographic differences. While the validation sample from WADRC closely resembles the initial WRAP sample in terms of demographics and region, the implications of including samples from different regions and diverse populations will be important for future research. The reproduction of our findings in an independent cohort further substantiates the credibility and applicability of our results.

### Limitations and future directions

A limitation of our study is the operationalization of cerebrovascular disease (V) through WMH alone, which although commonly used in ageing cohorts, may not fully capture the complexity of small vessel disease.^59^ In older adult populations, WMHs are often considered indicative of microvascular ischaemic lesions. However, other aspects of vascular pathology, such as microbleeds or alterations in cerebral haemodynamics (e.g. as observed on perfusion imaging), were not included in this study. These factors represent important areas for future investigation. We did not exclude infarcts or lacunar lesions, yet we still observe clear trajectories in WMH and no relationship with amyloid. We also believe this approach enhances the generalizability of our study. Also, the pattern and location of WMHs could provide further valuable insights into the relationship between vascular and amyloid pathology. Exploring the spatial relationships between WMHs and amyloid burden in future studies could deepen our understanding of how V and A do or do not interact in the brain. We used only one strategy to adjust WMH volume analyses for head size—total intracranial volume (TICV). Although it is common in research to normalize WMH volume to TICV to account for individual differences, the potential impact of small vessel disease (and WMHs) on brain volume suggests that alternative strategies may be warranted. Future studies should explore different approaches to determine whether the results hold. Additionally, while WMH burden has been used as a proxy for cerebrovascular disease, it is important to note that WMHs may be indicators of other diseases in different contexts,^60^ such as demyelinating diseases. Each MRI exam underwent neuroradiologist review, and cases resembling a demyelinating pattern in the judgement of the neuroradiologist were excluded; however, it remains possible that some cases were miscategorized as the range of imaging patterns does overlap. Furthermore, our analyses did not include diffusion imaging, which can reveal subtle changes in white matter before the appearance of hyperintensities.^61^ Future work may incorporate diffusion imaging with the goal of investigating the possibility of more nuanced connections between cerebrovascular pathology and amyloid accumulation.^62^ Additionally, we need to be clear about what we are discussing when interpreting the estimated V onset age for V− participants and the corresponding V+ chronicities in this group. Namely, while some in the V− category may have subthreshold WMH development that yield an accurate estimate of time to reaching the V+ threshold, the majority in the V− group are truly free of WMH’s and the slope and error estimates for the V− group (whether using age or V− chronicity as the time scale) provide insight into normal longitudinal variability associated with measurement error in those without WMH abnormalities. The consistency of slope estimates of annual change across various time operationalizations suggests that our estimation of V trajectories from the chronicitiy scale is reasonable for the V− group. Additionally, Fig. 1 shows some subjects with declining WMH burden. Perhaps technical limitations or variability in sources of the WMH signal could explain this, though physiologic improvement cannot be ruled out. Finally, there is emerging evidence linking WMH and tau,^63,64^ another key protein involved in Alzheimer’s disease. Including tau in these analyses was beyond the scope of our aims but will be important to address in the context of longitudinal analysis.

## Conclusion

There are three major conceptual advances in the present work: The demonstration of a consistent pattern of WMH accumulation in the V+ subset, evidence of temporal independence between A+ and V+ biomarkers over longitudinal observation and relatedly, that the presence of one does not perturb the progression of the other. These conclusions are highly consistent with Cogswell *et al.*^9^ and together provide important direction for the field. On the matter of cognitive decline in these pre-clinical late-middle age to older adult cohorts, we conclude, as have others, that the presence of both are deleterious and certainly both are worthwhile candidates for intervention in the interest of preventing or slowing clinical progression. The distinction in disease processes that we have articulated is crucial for applying future targeted therapies. While therapies focused on reducing CVD risk factors may diminish V-related imaging changes and associated cognitive decline, altering the course of cerebrovascular disease would not be expected to directly influence Alzheimer’s disease-related changes, such as amyloid accumulation. The testing of this hypothesis will need to be left for future work where an intervention for each is administered, a framework which is now possible with the advent of effective amyloid-lowering therapy.^65,66^

## Supplementary Material

fcaf158_Supplementary_Data

## Data Availability

Data from the WRAP (https://wrap.wisc.edu) can be requested through an online submission process. SILA algorithms are available at https://github.com/Betthauser-Neuro-Lab/SILA-AD-Biomarker.

## References

[1] McKhann GM, Knopman DS, Chertkow H, et al The diagnosis of dementia due to Alzheimer’s disease: Recommendations from the national institute on aging-Alzheimer’s association workgroups on diagnostic guidelines for Alzheimer’s disease. Alzheimers Dement. 2011;7(3):263–269. 21514250 10.1016/j.jalz.2011.03.005 PMC3312024

[2] Jack CR Jr, Bennett DA, Blennow K, et al NIA-AA research framework: Toward a biological definition of Alzheimer’s disease. Alzheimers Dement. 2018;14(4):535–562. 29653606 10.1016/j.jalz.2018.02.018 PMC5958625

[3] Jack CR Jr, Knopman DS, Jagust WJ, et al Tracking pathophysiological processes in Alzheimer’s disease: An updated hypothetical model of dynamic biomarkers. Lancet Neurol. 2013;12(2):207–216. 23332364 10.1016/S1474-4422(12)70291-0 PMC3622225

[4] Jack CR Jr, Bennett DA, Blennow K, et al A/T/N: An unbiased descriptive classification scheme for Alzheimer disease biomarkers. Neurology. 2016;87(5):539–547. 27371494 10.1212/WNL.0000000000002923 PMC4970664

[5] Brickman AM, Zahodne LB, Guzman VA, et al Reconsidering harbingers of dementia: Progression of parietal lobe white matter hyperintensities predicts Alzheimer’s disease incidence. Neurobiol Aging. 2015;36(1):27–32. 25155654 10.1016/j.neurobiolaging.2014.07.019 PMC4268124

[6] Debette S, Markus HS. The clinical importance of white matter hyperintensities on brain magnetic resonance imaging: Systematic review and meta-analysis. BMJ. 2010;341:c3666. 20660506 10.1136/bmj.c3666 PMC2910261

[7] DeCarli C, Murphy DGM, Tranh M, et al The effect of white matter hyperintensity volume on brain structure, cognitive performance, and cerebral metabolism of glucose in 51 healthy adults. Neurology. 1995;45(11):2077–2084. 7501162 10.1212/wnl.45.11.2077

[8] DeCarli C, Miller BL, Swan GE, Reed T, Wolf PA, Carmelli D. Cerebrovascular and brain morphologic correlates of mild cognitive impairment in the national heart, lung, and blood institute twin study. Arch Neurol. 2001;58(4):643–647. 11295996 10.1001/archneur.58.4.643

[9] Frank B, Ally M, Tripodis Y, et al Trajectories of cognitive decline in brain donors with autopsy-confirmed Alzheimer disease and cerebrovascular disease. Neurology. 2022;98(24):e2454–e2464. 35444054 10.1212/WNL.0000000000200304 PMC9231841

[10] Wardlaw JM, Valdés Hernández MC, Muñoz-Maniega S. What are white matter hyperintensities made of? Relevance to vascular cognitive impairment. J Am Heart Assoc. 2015;4(6):e001140. 26104658 10.1161/JAHA.114.001140 PMC4599520

[11] Luo J, Ma Y, Agboola FJ, et al Longitudinal relationships of white matter hyperintensities and Alzheimer disease biomarkers across the adult life span. Neurology. 2023;101(2):e164–e177. 37202169 10.1212/WNL.0000000000207378 PMC10351551

[12] Shirzadi Z, Schultz SA, Yau WYW, et al Etiology of white matter hyperintensities in autosomal dominant and sporadic Alzheimer disease. JAMA Neurol. 2023;80(12):1353–1363. 37843849 10.1001/jamaneurol.2023.3618 PMC10580156

[13] Lee S, Viqar F, Zimmerman ME, et al White matter hyperintensities are a core feature of Alzheimer’s disease: Evidence from the dominantly inherited Alzheimer network. Ann Neurol. 2016;79(6):929–939. 27016429 10.1002/ana.24647 PMC4884146

[14] Lane CA, Barnes J, Nicholas JM, et al Associations between vascular risk across adulthood and brain pathology in late life: Evidence from a British birth cohort. JAMA Neurol. 2020;77(2):175–183. 31682678 10.1001/jamaneurol.2019.3774 PMC6830432

[15] Roseborough A, Ramirez J, Black SE, Edwards JD. Associations between amyloid β and white matter hyperintensities: A systematic review. Alzheimers Dement. 2017;13(10):1154–1167. 28322203 10.1016/j.jalz.2017.01.026

[16] Bilgel M, Bannerjee A, Shafer A, An Y, Resnick SM. Vascular risk is not associated with PET measures of Alzheimer’s disease neuropathology among cognitively normal older adults. Neuroimage Rep. 2021;1(4):100068. 34927122 10.1016/j.ynirp.2021.100068 PMC8682073

[17] Cogswell PM, Lundt ES, Therneau TM, et al Evidence against a temporal association between cerebrovascular disease and Alzheimer’s disease imaging biomarkers. Nat Commun. 2023;14(1):3097. 37248223 10.1038/s41467-023-38878-8 PMC10226977

[18] Kim SJ, Lee DK, Jang YK, et al The effects of longitudinal white matter hyperintensity change on cognitive decline and cortical thinning over three years. J Clin Med. 2020;9(8):2663. 32824599 10.3390/jcm9082663 PMC7465642

[19] Rizvi B, Lao PJ, Chesebro AG, et al Association of regional white matter hyperintensities with longitudinal Alzheimer-like pattern of neurodegeneration in older adults. JAMA Netw Open. 2021;4(10):e2125166. 34609497 10.1001/jamanetworkopen.2021.25166 PMC8493439

[20] Grimmer T, Faust M, Auer F, et al White matter hyperintensities predict amyloid increase in Alzheimer’s disease. Neurobiol Aging. 2012;33(12):2766–2773. 22410648 10.1016/j.neurobiolaging.2012.01.016

[21] Betthauser TJ, Bilgel M, Koscik RL, et al Multi-method investigation of factors influencing amyloid onset and impairment in three cohorts. Brain. 2022;145(11):4065–4079. 35856240 10.1093/brain/awac213 PMC9679170

[22] Bilgel M, Koscik RL, An Y, et al Temporal order of Alzheimer’s disease-related cognitive marker changes in BLSA and WRAP longitudinal studies. J Alzheimers Dis. 2017;59(4):1335–1347. 28731452 10.3233/JAD-170448 PMC5682938

[23] Koscik RL, Betthauser TJ, Jonaitis EM, et al Amyloid duration is associated with preclinical cognitive decline and tau PET. Alzheimers Dement (Amst). 2020;12(1):e12007. 32211502 10.1002/dad2.12007 PMC7085284

[24] Schindler SE, Li Y, Buckles VD, et al Predicting symptom onset in sporadic Alzheimer disease with amyloid PET. Neurology. 2021;97(18):e1823–e1834. 34504028 10.1212/WNL.0000000000012775 PMC8610624

[25] Therneau TM, Knopman DS, Lowe VJ, et al Relationships between β-amyloid and tau in an elderly population: An accelerated failure time model. NeuroImage. 2021;242:118440. 34333107 10.1016/j.neuroimage.2021.118440 PMC8499700

[26] Johnson SC, Koscik RL, Jonaitis EM, et al The Wisconsin registry for Alzheimer’s prevention: A review of findings and current directions. Alzheimers Dement. 2018;10(1):130–142. 10.1016/j.dadm.2017.11.007 PMC5755749 29322089

[27] Beekly DL, Ramos EM, Lee WW, et al The national Alzheimer’s coordinating center (NACC) database: The uniform data set. Alzheimer Dis Assoc Disord. 2007;21(3):249–258. 17804958 10.1097/WAD.0b013e318142774e

[28] Schmidt M . Rey auditory verbal learning test: A handbook. Vol 17. Western Psychological Services; 1996.

[29] Donohue MC, Sperling RA, Petersen R, et al Association between elevated brain amyloid and subsequent cognitive decline among cognitively normal persons. JAMA. 2017;317(22):2305–2316. 28609533 10.1001/jama.2017.6669 PMC5736301

[30] Wechsler D . Wechsler memory scale-revised. Psychological Corporation; 1987.

[31] Wechsler D . WAIS-III: Wechsler adult intelligence scale. Psychological Corporation; 1997.

[32] Monsell SE, Dodge HH, Zhou XH, et al Results from the NACC uniform data set neuropsychological battery crosswalk study. Alzheimer Dis Assoc Disord. 2016;30(2):134–139. 26485498 10.1097/WAD.0000000000000111 PMC4834278

[33] Weintraub S, Besser L, Dodge HH, et al Version 3 of the Alzheimer disease centers’ neuropsychological test battery in the uniform data set (UDS). Alzheimer Dis Assoc Disord. 2018;32(1):10–17. 29240561 10.1097/WAD.0000000000000223 PMC5821520

[34] Reitan RM . Validity of the trail making test as an indicator of organic brain damage. Percept Mot Skills. 1958;8(3):271–276.

[35] Jonaitis EM, Hermann BP, Mueller KD, et al Longitudinal normative standards for cognitive tests and composites using harmonized data from two Wisconsin AD-risk-enriched cohorts. Alzheimers Dement. 2024;20(5):3305–3321. 38539269 10.1002/alz.13774 PMC11095443

[36] Jonaitis EM, Koscik RL, Clark LR, et al Measuring longitudinal cognition: Individual tests versus composites. Alzheimers Dement (Amst). 2019;11:74–84. 31673596 10.1016/j.dadm.2018.11.006 PMC6816509

[37] Clark LR, Koscik RL, Nicholas CR, et al Mild cognitive impairment in late middle age in the Wisconsin registry for Alzheimer’s prevention study: Prevalence and characteristics using robust and standard neuropsychological normative data. Arch Clin Neuropsychol. 2016;31(7):675–688. 27193363 10.1093/arclin/acw024 PMC5088607

[38] Du L, Koscik RL, Chin NA, et al Prescription medications and co-morbidities in late middle-age are associated with greater cognitive declines: Results from WRAP. Front Aging. 2022;2:759695. 35822000 10.3389/fragi.2021.759695 PMC9261362

[39] Langhough Koscik R, Hermann BP, Allison S, et al Validity evidence for the research category, “cognitively unimpaired–declining,” as a risk marker for mild cognitive impairment and Alzheimer’s disease. Front Aging Neurosci. 2021;13:688478. 34381351 10.3389/fnagi.2021.688478 PMC8350058

[40] Albert MS, DeKosky ST, Dickson D, et al The diagnosis of mild cognitive impairment due to Alzheimer’s disease: Recommendations from the national institute on aging-Alzheimer’s association workgroups on diagnostic guidelines for Alzheimer’s disease. Alzheimers Dement. 2011;7(3):270–279. 21514249 10.1016/j.jalz.2011.03.008 PMC3312027

[41] Johnson SC, Christian BT, Okonkwo OC, et al Amyloid burden and neural function in people at risk for Alzheimer’s disease. Neurobiol Aging. 2014;35(3):576–584. 24269021 10.1016/j.neurobiolaging.2013.09.028 PMC4018215

[42] Scrucca L, Fop M, Murphy TB, Raftery AE. Mclust 5: Clustering, classification and density estimation using Gaussian finite mixture models. R J. 2016;8(1):289–317. 27818791 PMC5096736

[43] Sprecher KE, Bendlin BB, Racine AM, et al Amyloid burden is associated with self-reported sleep in non-demented late middle-aged adults. Neurobiol Aging. 2015;36(9):2568–2576. 26059712 10.1016/j.neurobiolaging.2015.05.004 PMC4523445

[44] Racine AM, Clark LR, Berman SE, et al Associations between performance on an abbreviated CogState battery, other measures of cognitive function, and biomarkers in people at risk for Alzheimer’s disease. J Alzheimers Dis. 2016;54(4):1395–1408. 27589532 10.3233/JAD-160528 PMC5074904

[45] R Core Team . R: A language and environment for statistical computing. R Foundation for Statistical Computing; 2020.

[46] Lenth RV, Bolker B, Buerkner P, et al emmeans: Estimated marginal means, aka least-squares means. Comprehensive R Archive Network (CRAN) Accessed 20 March 2025. https://cran.r-project.org/web/packages/emmeans/index.html. 2023.

[47] Burnham KP, Anderson DR, Huyvaert KP. AIC model selection and multimodel inference in behavioral ecology: Some background, observations, and comparisons. Behav Ecol Sociobiol. 2011;65(1):23–35.

[48] Kapasi A, Yu L, Petyuk V, Arfanakis K, Bennett DA, Schneider JA. Association of small vessel disease with tau pathology. Acta Neuropathol. 2022;143(3):349–362. 35044500 10.1007/s00401-021-02397-x PMC8858293

[49] McAleese KE, Miah M, Graham S, et al Frontal white matter lesions in Alzheimer’s disease are associated with both small vessel disease and AD-associated cortical pathology. Acta Neuropathol. 2021;142(6):937–950. 34608542 10.1007/s00401-021-02376-2 PMC8568857

[50] McAleese KE, Walker L, Graham S, et al Parietal white matter lesions in Alzheimer’s disease are associated with cortical neurodegenerative pathology, but not with small vessel disease. Acta Neuropathol. 2017;134(3):459–473. 28638989 10.1007/s00401-017-1738-2 PMC5563333

[51] Graff-Radford J, Arenaza-Urquijo EM, Knopman DS, et al White matter hyperintensities: Relationship to amyloid and tau burden. Brain. 2019;142(8):2483–2491. 31199475 10.1093/brain/awz162 PMC6658846

[52] Lorenzini L, Ansems LT, Lopes Alves I, et al Regional associations of white matter hyperintensities and early cortical amyloid pathology. Brain Commun. 2022;4(3):fcac150. 35783557 10.1093/braincomms/fcac150 PMC9246276

[53] Pillai SKR, Reid RI, Przybelski SA, et al Diffusion models reveal white matter microstructural changes with aging, pathology, and cognition. Alzheimers Dement. 2021;17(S4):e053109. 10.1093/braincomms/fcab106 PMC8202149 34136811

[54] Koncz R, Thalamuthu A, Wen W, et al The heritability of amyloid burden in older adults: The older Australian twins study. J Neurol Neurosurg Psychiatry. 2022;93(3):303–308. 34921119 10.1136/jnnp-2021-326677

[55] Pettigrew C, Soldan A, Wang J, et al Association of midlife vascular risk and AD biomarkers with subsequent cognitive decline. Neurology. 2020;95(23):e3093–e3103. 32989109 10.1212/WNL.0000000000010946 PMC7734925

[56] Vemuri P, Lesnick TG, Przybelski SA, et al Vascular and amyloid pathologies are independent predictors of cognitive decline in normal elderly. Brain. 2015;138(Pt 3):761–771. 25595145 10.1093/brain/awu393 PMC4339775

[57] Marchant NL, Reed BR, DeCarli CS, et al Cerebrovascular disease, β-amyloid, and cognition in aging. Neurobiol Aging. 2012;33(5):1006.e25–1006.e36. 10.1016/j.neurobiolaging.2011.10.001 PMC3274647 22048124

[58] Rosano C, Aizenstein HJ, Wu M, et al Focal atrophy and cerebrovascular disease increase dementia risk among cognitively normal older adults. J Neuroimaging. 2007;17(2):148–155. 17441836 10.1111/j.1552-6569.2007.00093.x

[59] Wardlaw JM, Smith C, Dichgans M. Small vessel disease: Mechanisms and clinical implications. Lancet Neurol. 2019;18(7):684–696. 31097385 10.1016/S1474-4422(19)30079-1

[60] Garnier-Crussard A, Cotton F, Krolak-Salmon P, Chételat G. White matter hyperintensities in Alzheimer’s disease: Beyond vascular contribution. Alzheimers Dement. 2023;19(8):3738–3748. 37027506 10.1002/alz.13057

[61] Moody JF, Dean DC, Kecskemeti SR, et al Associations between diffusion MRI microstructure and cerebrospinal fluid markers of Alzheimer’s disease pathology and neurodegeneration along the Alzheimer’s disease continuum. Alzheimers Dement (Amst). 2022;14(1):e12381. 36479018 10.1002/dad2.12381 PMC9720004

[62] Araque Caballero MÁ, Suárez-Calvet M, Duering M, et al White matter diffusion alterations precede symptom onset in autosomal dominant Alzheimer’s disease. Brain. 2018;141(10):3065–3080. 30239611 10.1093/brain/awy229 PMC6158739

[63] Rahmani F, Brier MR, Gordon BA, et al T1 and FLAIR signal intensities are related to tau pathology in dominantly inherited Alzheimer disease. Hum Brain Mapp. 2023;44(18):6375–6387. 37867465 10.1002/hbm.26514 PMC10681640

[64] Alban SL, Lynch KM, Ringman JM, et al The association between white matter hyperintensities and amyloid and tau deposition. Neuroimage Clin. 2023;38:103383. 36965457 10.1016/j.nicl.2023.103383 PMC10060905

[65] Sims JR, Zimmer JA, Evans CD, et al Donanemab in early symptomatic Alzheimer disease: The TRAILBLAZER-ALZ 2 randomized clinical trial. JAMA. 2023;330(6):512–527. 37459141 10.1001/jama.2023.13239 PMC10352931

[66] van Dyck CH, Swanson CJ, Aisen P, et al Lecanemab in early Alzheimer’s disease. N Engl J Med. 2023;388(1):9–21. 36449413 10.1056/NEJMoa2212948

