# Supplementary material for: Onset ages of cerebrovascular disease and amyloid and effects on cognition in risk-enriched cohorts
Source: Brain Commun. 2025 Apr 19;7(3):fcaf158. doi: 10.1093/braincomms/fcaf158 (PMC12056727; doi:10.1093/braincomms/fcaf158)
Supplement: fcaf158_Supplementary_Data [file fcaf158_supplementary_data.docx]

**Supplementary Figures and Tables to the manuscript “Onset ages of cerebrovascular disease and amyloid and effects on cognition in risk-enriched cohorts”**

**Running title:** Cerebrovascular, amyloid and cognition

Lianlian Du^1,3,4,5*^, Elizabeth M. Planalp^1,2,3^, Tobey J. Betthauser^2,3^, Erin M. Jonaitis^1,2,3^, Bruce P. Hermann^1,6^, Leonardo A. Rivera-Rivera^2,3,7^, Karly A. Cody^2,8^, Nathaniel A. Chin^2,3^, Robert V. Cadman^2,3^, Kevin M. Johnson^7,9^, Aaron Field^2,9^, Howard A. Rowley^2,9^, Kimberly D Mueller^1,2,3,10^, Sanjay Asthana^2,3^, Laura Eisenmenger^9^, Bradley T. Christian^7^, Sterling C. Johnson^1,2,3^, Rebecca E. Langhough^1,2,3*^

**Affiliations**

1 Wisconsin Alzheimer’s Institute, University of Wisconsin-Madison School of Medicine and Public Health, Madison, WI, 53792, USA

2 Wisconsin Alzheimer’s Disease Research Center, Madison, WI, 53792, USA

3 Department of Medicine, University of Wisconsin-Madison School of Medicine and Public Health, Madison, WI, 53792, USA

4 Rush Alzheimer’s Disease Center, Rush University Medical Center; Chicago, IL, USA

5 Department of Neurological Sciences, Rush University Medical Center; Chicago, IL, USA

6 Department of Neurology, University of Wisconsin-Madison School of Medicine and Public Health, Madison, WI, 53792, USA

7 Department of Medical Physics, University of Wisconsin-Madison School of Medicine and Public Health, Madison, WI, 53792, USA

8 Stanford University, Department of Neurology and Neurological Sciences, Palo Alto, CA, 94304, USA

9 Department of Radiology, University of Wisconsin School of Medicine and Public Health, Madison, WI, 53792, USA

10 Department of Communication Sciences and Disorders, University of Wisconsin-Madison, Madison, WI, 53792, USA

*Correspondence to: Rebecca Langhough, Wisconsin Alzheimer’s Institute, University of Wisconsin-Madison, School of Medicine and Public Health, 600 Highland Avenue, K6/430, Clinical Sciences Center, Madison, WI, 53792, USA.

[langhough@wisc.edu](mailto:rekoscik@wisc.edu)

Lianlian Du, Department of Medicine, School of Medicine and Public Health, University of Wisconsin-Madison, 600 Highland Avenue, K6/446, Clinical Sciences Center, Madison, WI, 53792, USA.

[ldu39@wisc.edu](mailto:ldu@medicine.wisc.edu)

## **eMethods and eResults**

### Amyloid and WMH Trajectory Modeling Methods

#### Sampled Iterative Local Approximation

The Sampled Iterative Local Approximation (SILA) algorithm uses a three-step process that includes discrete sampling of biomarker accumulation rates, smoothing, and numerical integration to generate a nonparametric amyloid vs. time curve. Individual estimates of A+ duration and age of A+ onset are obtained by solving the nonparametric curve for the duration of A+ corresponding to a given DVR or SUVR value at one or more PET observations.

The first step in the modeling algorithm applies discrete sampling to within-person annualized longitudinal DVR or SUVR slopes to establish the ‘observed’ relationship between the annualized amyloid accumulation rate and amyloid burden. Query DVR or SUVR values are established by dividing the full range of observed values into 150 equally spaced values. For each query value, the mean SUVR or DVR rate is calculated for participants whose observations intersect the query value.

The second step of the algorithm optimizes a robust weighted local second-degree polynomial smoothing kernel (rloess option in MATLAB’s smooth function) applied to the numerical rate vs. level data by minimizing the weighted sum of squared backward prediction residuals (i.e., predicting the first DVR or SUVR value using the last observation as a reference). A weighting function is applied to optimize the kernel size by de-weighting the sum of squares for A- cases, multiplying them by the ratio of A+ to A- cases. This prioritizes A+ cases for the modeled amyloid vs. time function.

The third step uses Euler’s method to numerically integrate the smoothed amyloid rate vs. amyloid level function to produce an amyloid level vs. time curve. For this study, a 0.25-year step size was used for numerical integration, with the initial condition that the DVR or SUVR positivity threshold corresponds to time = 0 years. Thus, the time axis corresponds to the number of years a person has been A+. The algorithm terminates if either the number of observations for a given amyloid level is less than two, the average slope is ≤ 0, or the maximum iteration limit of 200 (corresponding to 50 years) is reached.

A+ duration is estimated for individual participants at a reference observation (single scan) by numerically solving the nonparametric amyloid vs. A+ duration function for time, inputting the observed DVR or SUVR value at the reference scan. The age of A+ onset is then estimated by subtracting the estimated A+ duration from the age at the reference scan. The algorithm also includes a within-person least squares option to solve for A+ time using multiple observations, but this was not used in the current study. Linear extrapolation of the first or last three years of the modeled curve was used to estimate age and duration of A+ if reference values were out of range of the modeled nonparametric curve. The duration of A+ at the reference scan for cases whose observations were below the modeled DVR or SUVR range was truncated so that the reference scan for each participant was aligned to the earliest modeled point on the nonparametric amyloid vs. time curve.

Forward and backward DVR or SUVR predictions were accomplished by solving the amyloid vs. time curve for DVR or SUVR corresponding to the duration of A+ at the time of the reference scan minus the time of the target scan being predicted.

This approach has been applied to longitudinal amyloid trajectories, where the algorithm estimates both A+ duration and age of A+ onset for each individual. The method has been implemented in previous studies, such as Betthauser et al., 2022,^1^ and Zammit et al., 2023.^2^ Details about the SILA method can be found here: <https://github.com/Betthauser-Neuro-Lab/SILA-AD-Biomarker>. This study is the first to implement the SILA method to characterize the WMH longitudinal trajectory. The method is the same as described above, except that the threshold is based on the Gaussian Mixture Model.

### Comparing slopes of the V- set using multiple time operationalizations

While slopes based on chronicities in the V+ group are thought to provide more accurate estimates of annualized change and variability in those who have WMH abnormalities than slopes based on other time operationalizations (such as age or time since last MRI), slopes based on chronicities in the V- group offer another means of estimation of measurement variability in those who do not have evidence of white matter hyperintensity accumulation while allowing statistically more powerful direct comparison of biomarker negative and positive time-related changes. Since the chronicities assigned to V- are not actually thought to indicate time to V+ and given that the V+ group is older than the V- group, the chronicity time scale may introduce some bias in estimates of annualized change.

We investigated evidence for our assumption that chronicity-based slope estimates in the V- group will be consistent with those using other operationalizes of time in parallel LME models as follows. First, we operationalized time 3 ways in the V- subset: 1) age in years at the time of MRI, 2) years before last MRI scan, and 3) to simulate the same chronicity range as the V+ group, we randomly selected a last chronicity from the V+ group for each V- participant and used that to estimate chronicity. Then, in lme models, we compared slopes across each operationalization. For all, we limited the V- group to the subset that had a chronicity of <-3 years at last scan. This value was selected based on work in our group and others that shows a phase of 1-5 years between onset of accumulation and reaching a positive threshold. Supplementary Figure 4, panels A-C shows spaghetti plots of observed WMH data vs each of the time operationalizations with the time(se) model estimate superimposed on each. Supplementary Figure 4, panel D shows the forest plot of these slope estimates: centered age, years to the last MRI, and chronicity based on randomly selected V+ chronicity. We repeated the analysis, restricting the age range to 60-80. Supplementary Figure 4, panel D also shows the slope estimates for this subset.

As Supplementary Figure 4D shows, the beta(se) estimates of annual change in WMH for this subset from the three models overlap. The annual change in WMH (V) and the slope of the V- group were similar to those of the whole group, across all time operationalizations. Therefore, these are valid estimates of longitudinal change in people who are negative for the biomarker, or comparisons of rates of change with those who are biomarker positive.

# **References**

1. Betthauser TJ, Bilgel M, Koscik RL, et al. Multi-method investigation of factors influencing amyloid onset and impairment in three cohorts. *Brain*. 2022;145(11):4065-4079. doi:10.1093/brain/awac213

2. Zammit MD, Betthauser TJ, McVea AK, et al. Characterizing the emergence of amyloid and tau burden in Down syndrome. *Alzheimers Dement*. 2024;20(1):388-398.


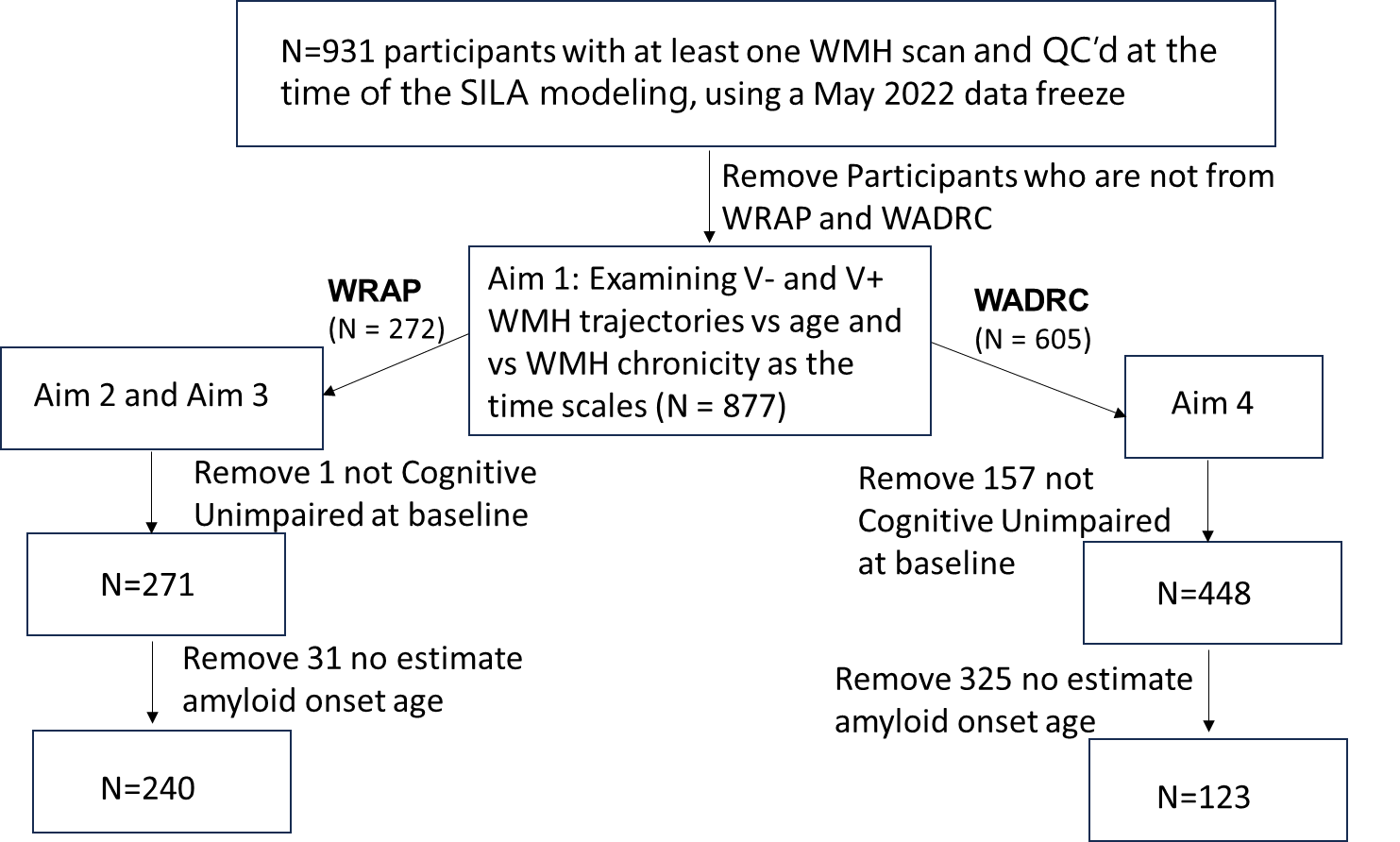


**Supplementary Fig. 1. Flowchart of Participant Inclusion Criteria for Each Aim.**

The flowchart outlines the participant selection process for each aim. The initial sample included N = 931, with exclusions based on predefined criteria. The final analytical sample sizes were N = 877 for Aim 1, N = 240 for Aim 2 and Aim 3, and N = 123 for Aim 4.

**
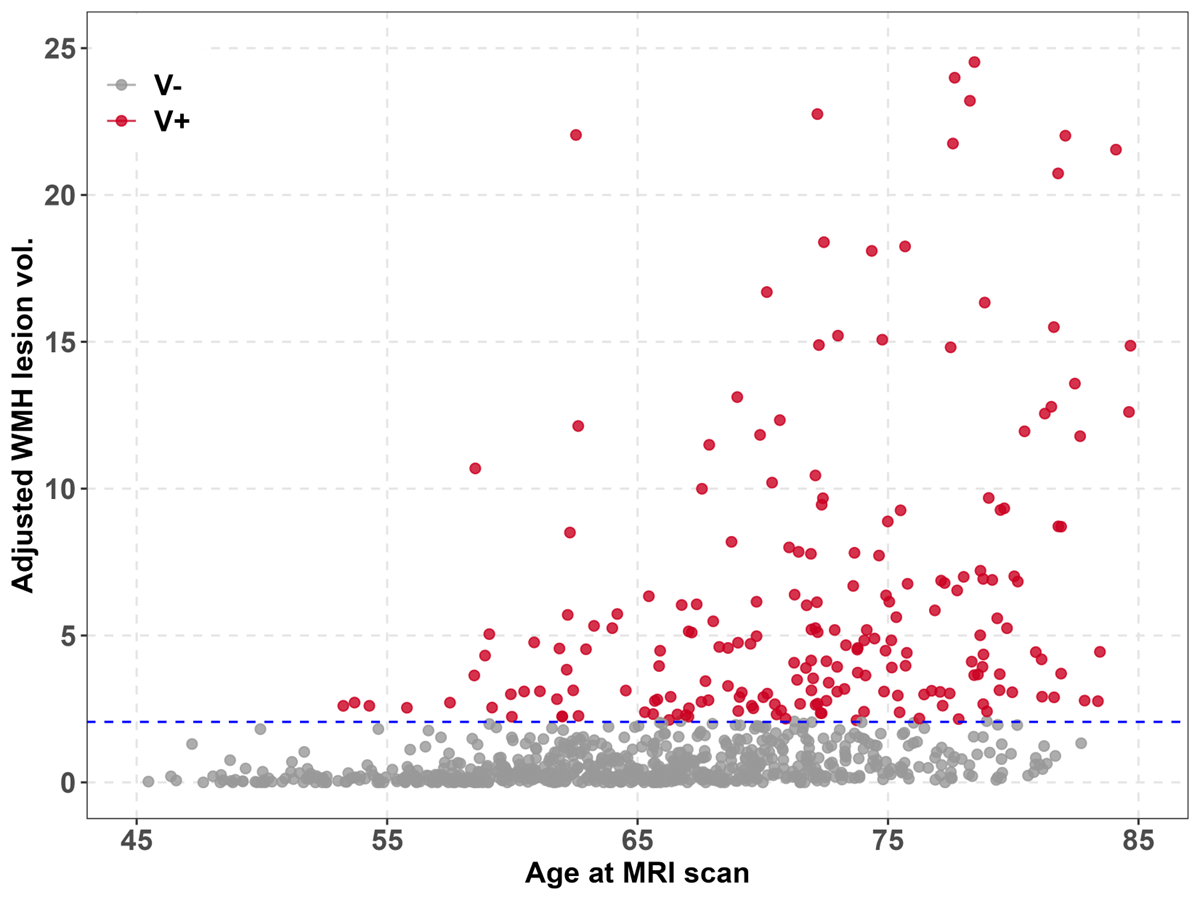
**

**Supplementary Fig. 2. The adjusted WMH volume threshold from GMM on most recent data (n=931).**

The blue dashed lines are the threshold from GMM (adjusted WMH ≥2.06 mL indicates V+). In GMM analysis, the algorithm estimates parameters such as means, variances, and mixing proportions for each component of the mixture distribution. By fitting GMM to the data, we identified distinct clusters representing different patterns of WMH volumes (BIC = -4666.752). To decide the threshold, we considered the adjusted WMH volume corresponding to the intersection of the probability density functions of the two classes. This threshold value served as the cutoff point to classify WMH volumes as V+ or V-. Abbreviations: V = White Matter Hyperintensities, GMM = Gaussian Mixture Model.

**
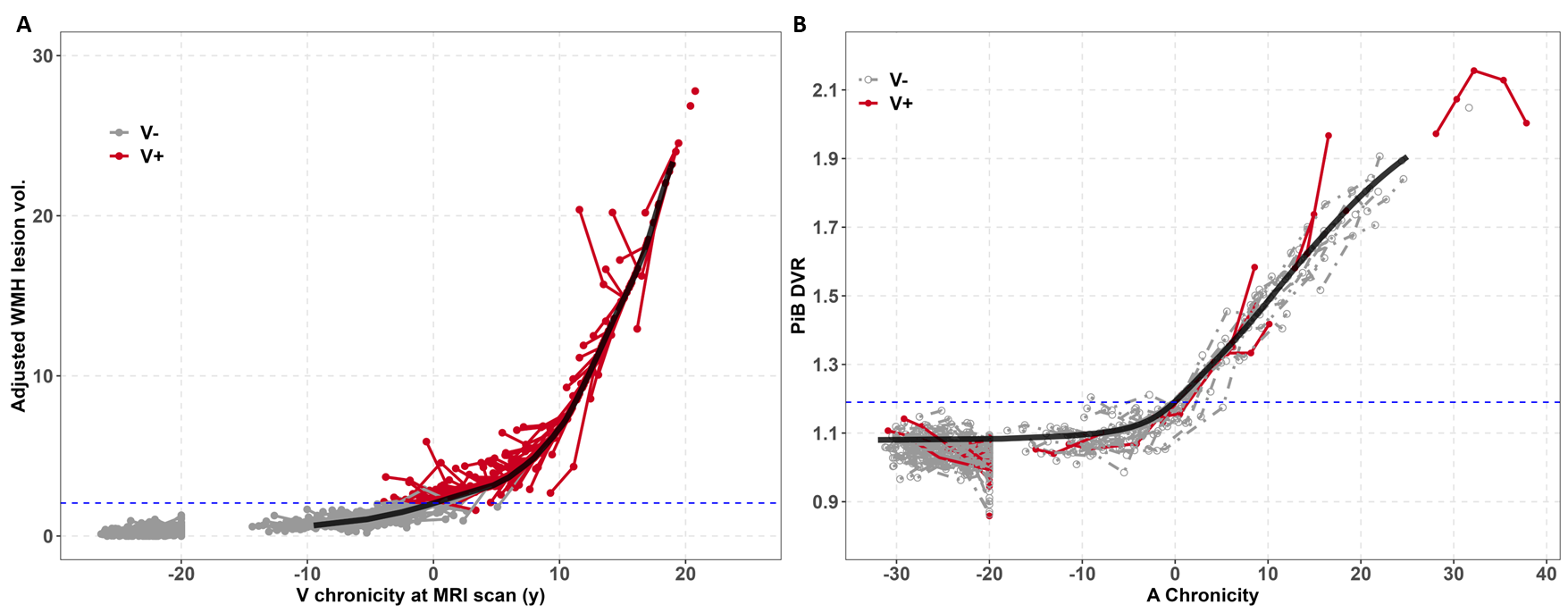
**

**Supplementary Fig. 3. The SILA-modeled curves on WMH lesion volume (n=877) and PiB DVR (n=240).**

The black lines are SILA modeled curves. The blue dashed lines are the threshold we used in this paper. A: The spaghetti plot of WMH vs. V chronicity, by V+/- status (Aim1, Figure 1B). B: The spaghetti plot of PiB DVR vs. A chronicity by V+/- status (Aim 2b, Figure 3B). Abbreviations: SILA = Sampled iterative local approximation, WMH/V = White Matter Hyperintensities, PiB = Pittsburgh Compound-B, DVR = Distribution Volume Ratio.

**
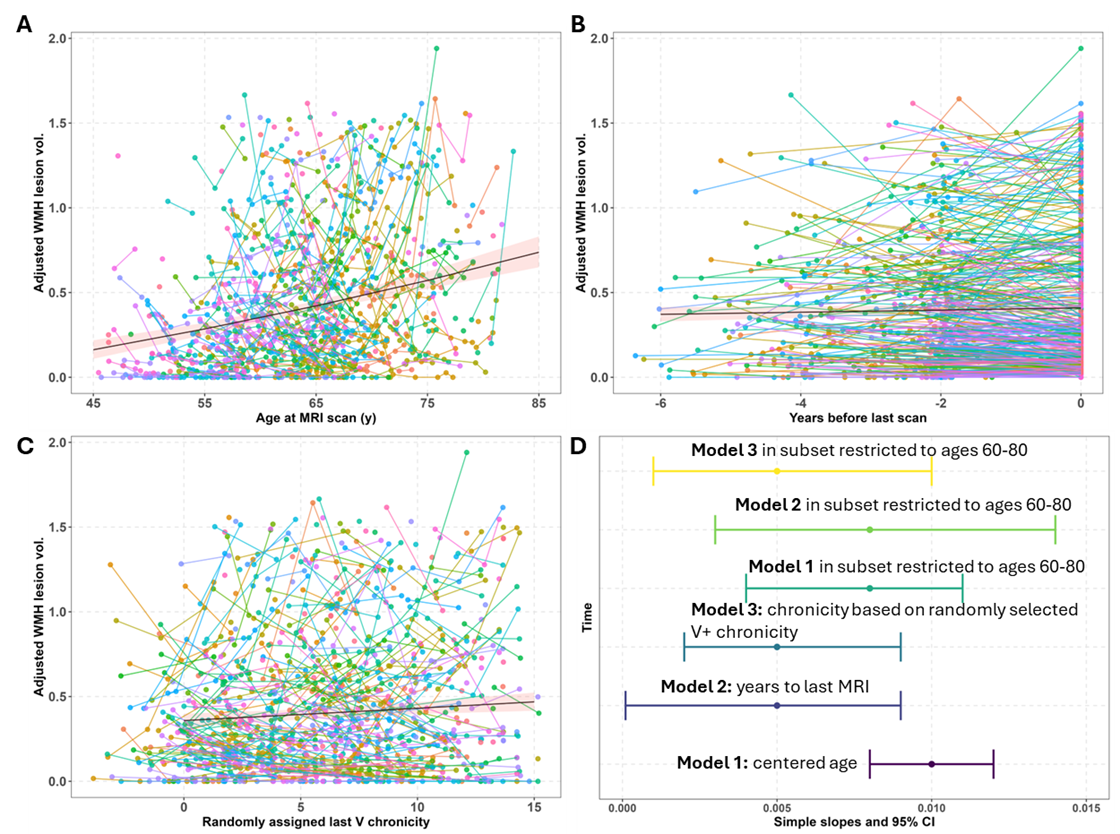
**

**Supplementary Fig. 4. The spaghetti plot of WMH lesion volume vs three additional time operationalizations and forest plot of slope estimates (n=611, V- subset).**

Panels A-C shows spaghetti plots of observed WMH data vs each of the time operationalizations with the time(se) model estimate superimposed on each (age beta CI=0.008-0.012, p < 0.001; years before last scan beta CI=0.0001-0.009, p = 0.005; chronicity beta CI=0.002-0.009, p =0.003). Panel D shows the forest plot of these slope estimates from Model 1 (Panel A), Model 2 (Panel B), and Model 3 (Panel C), as well as the same model set applied to the subset restricted to ages 60–80. Patterns suggest that slope estimates based on age may be more influenced by those who are accumulating white matter hyperintensities but have not yet reached the WMH+ threshold that was established using GMM.

**
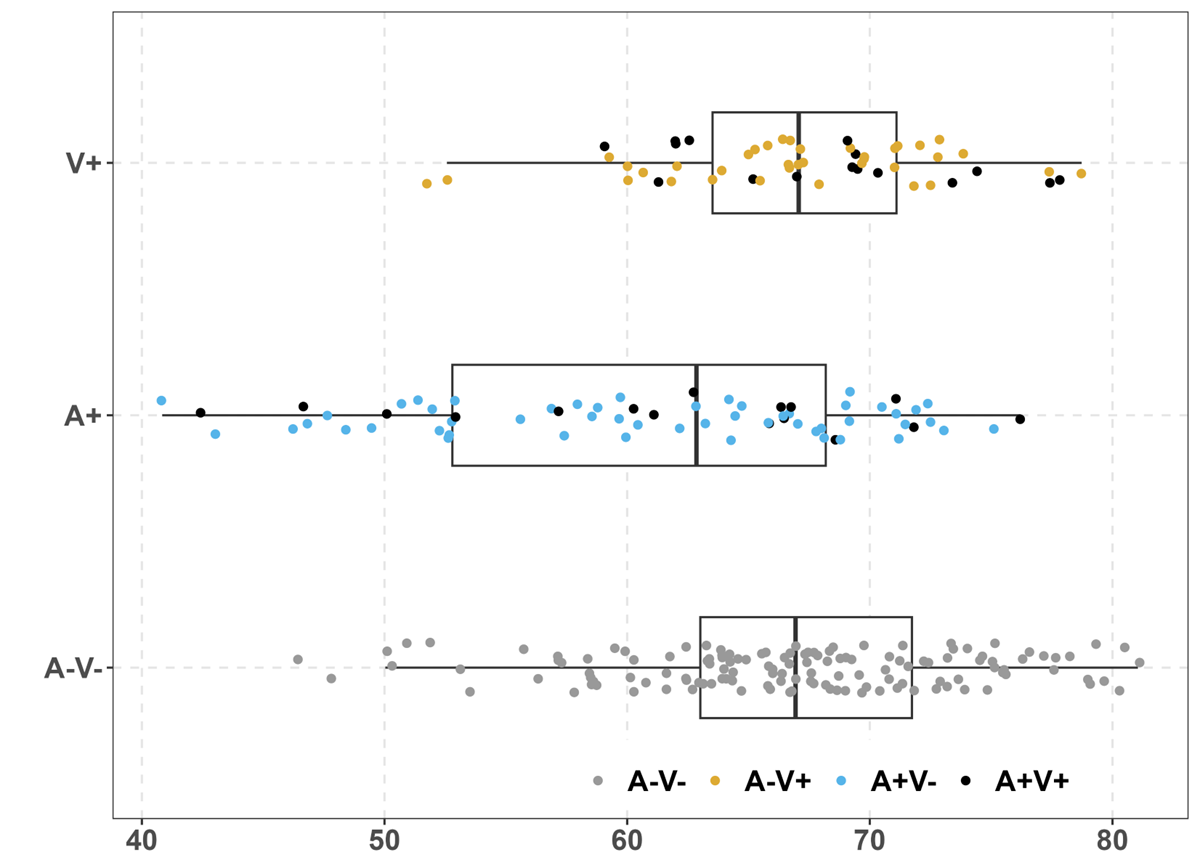
**

**Supplementary Fig. 5. The boxplots of SILA-estimated onset ages in A+ V+ and right censored ages in A-V- group (n=240; WRAP).**

Abbreviations: SILA = Sampled iterative local approximation, V = White Matter Hyperintensities, A = Amyloid PET.


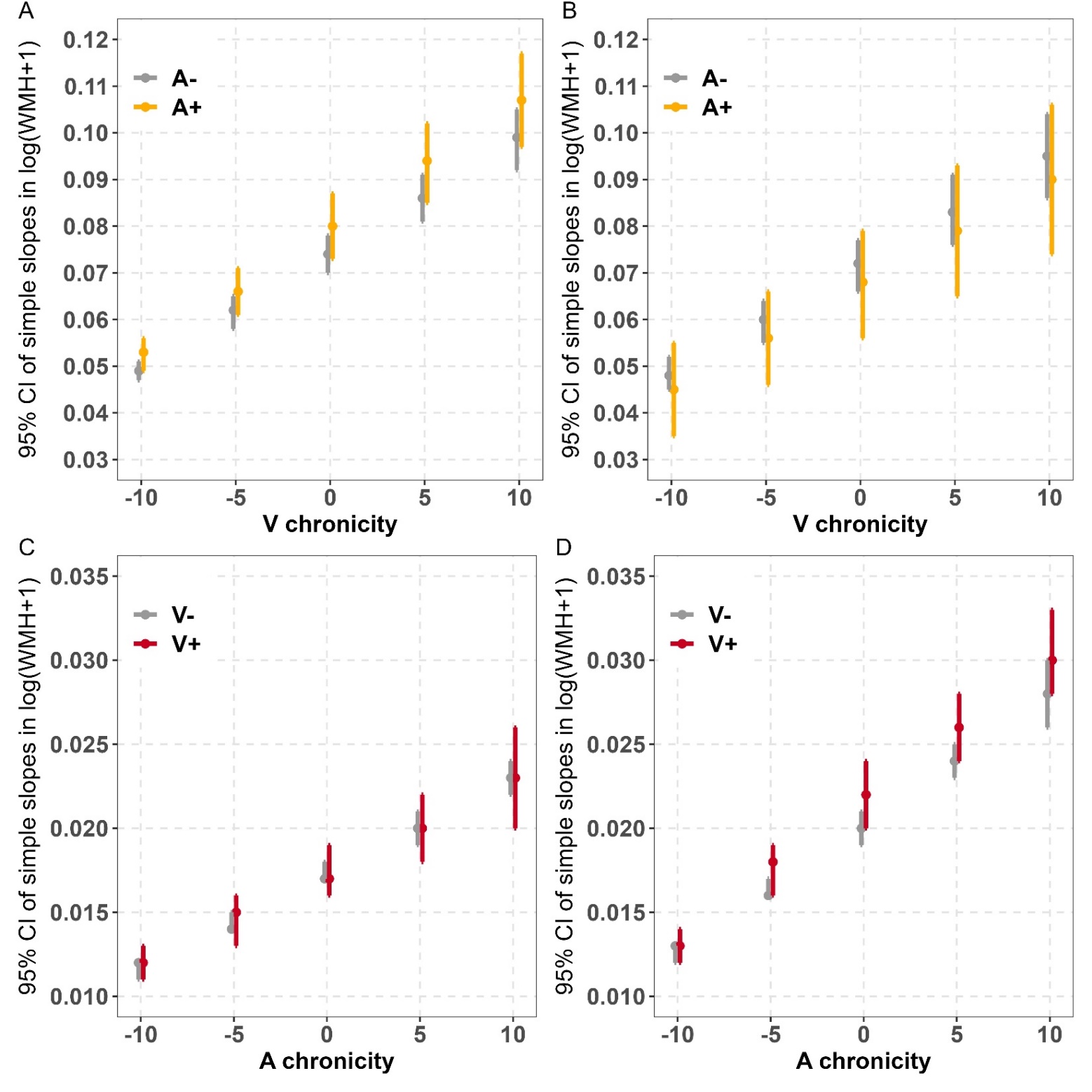
**Supplementary Fig. 6. Simple slope (95% CI’s) estimated from “model 2” mixed effects models in aim 2a and 2b run separately for the biomarker subgroups shown (A&C: n=240, WRAP; B&D: n = 123, WADRC).**

Panels A (A-: V chronicity beta CI=0.07 – 0.08, p < 0.001, V chronicity ^2 beta CI=0.00 – 0.00, p < 0.001; A+: V chronicity beta CI=0.07 – 0.09, p < 0.001, V chronicity ^2 beta CI=0.00 – 0.00, p < 0.001) and B (A-: V chronicity beta CI=0.07 – 0.08, p < 0.001, V chronicity ^2 beta CI=0.00 – 0.00, p < 0.001; A+: V chronicity beta CI=0.06 – 0.08, p < 0.001, V chronicity ^2 beta CI=0.00 – 0.00, p < 0.001) depict estimated annual change in log(WMH lesion volume) in WRAP and WADRC, respectively, while Panels C (V-: A chronicity beta CI=0.02 – 0.02, p < 0.001, A chronicity ^2 beta CI=0.00 – 0.00, p < 0.001; V+: A chronicity beta CI=0.02 – 0.02, p < 0.001, A chronicity ^2 beta CI=0.00 – 0.00, p < 0.001) and D (V-: A chronicity beta CI=0.02 – 0.02, p < 0.001, A chronicity ^2 beta CI=0.00 – 0.00, p < 0.001; V+: A chronicity beta CI=0.02 – 0.02, p < 0.001, A chronicity ^2 beta CI=0.00 – 0.00, p < 0.001) depict estimated annual change in global PiB DVR in WRAP and WADRC, respectively.

**
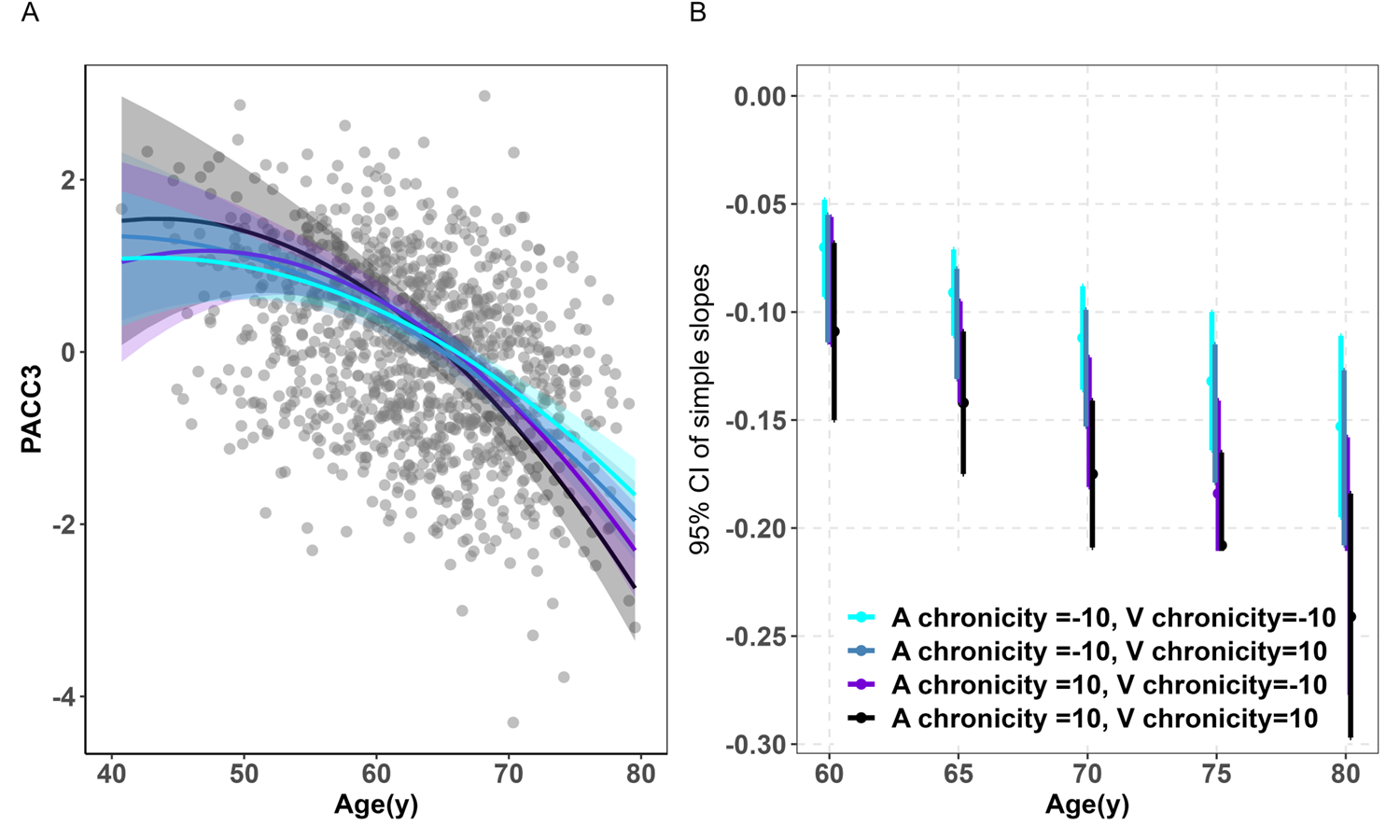
**

**Supplementary Fig. 7.Observed PACC3 z-scores (dots) and simple slopes for median of negative and positive range of each biomarker in sensitivity analysis of aim 3.** .

A: Observed PACC3 z-scores (dots) and interaction plots for four groups defined by the median of negative and positive ranges of A chronicity and V chronicity (−10 and +10). B: Simple slopes for the same four groups (based on A and V chronicity at −10 or +10) at ages 60, 65, 70, 75, and 80. Model was PACC3 z-scores ~ c60_age * PiB chronicity *V chronicity + c60_age * PiB chronicity + c60_age * V chronicity + V chronicity * PiB chronicity + quadratic c60_age * PiB chronicity + Sex + WRAT3 + education + number of prior exposures + random person‐level intercepts and age slopes; c60_age indicates age centered at 60 (age* A chronicity * V chronicity beta CI=-0.02 – -0.00, p =0.048). Abbreviations: WMH = White Matter Hyperintensities, PACC3 = Preclinical Alzheimer’s Cognitive Composite.


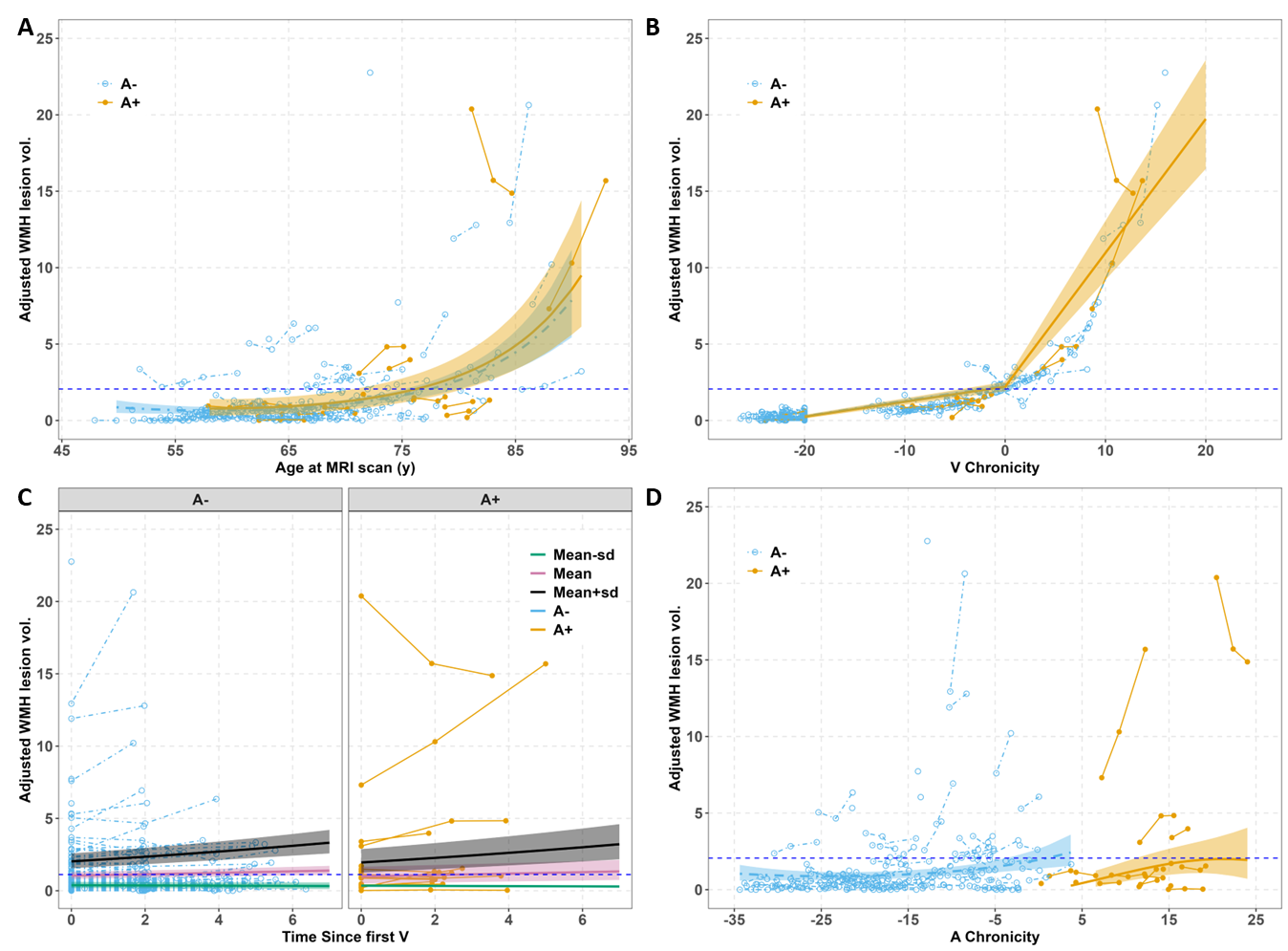


**Supplementary Fig. 8. Aim 4 replicated Aim 2a WMH spaghetti plots and predicted slopes from mixed effects for four time operationalizations (WADRC; n=123).**

A. WMH vs age, by A+/- status. Trajectories show WMH typically begins later in life. Simple slopes analyses indicate that A+ and A- WMH slopes did not differ significantly (A status beta CI=-0.23-0.38, p = 0.614). B. WMH vs SILA-aligned V+ chronicity (labeled “Years V+”) at time of each scan (calculated as age at scan minus V+ onset age). No significant difference between A+ and A- WMH trajectories. Note: To include those who are V- in the visualization, “Years V+” was truncated to -20 for last scan of those who are V-. C. Simple slopes plot of significant baseline WMH age*A status*years since baseline WMH interaction (baseline age* time since first V beta CI=0.00-0.00, p = < 0.001). Baseline WMH ages depicted are: 57.3 (mean-1SD), 65.8 (mean), and 74.3 (mean+1SD). Patterns suggest WMH increases faster in A+ vs A- in those who are older but not in those who are younger. D. WMH vs A+ chronicity at WMH scans (A status * A chronicity beta CI=0.00-(0.16), p = 0.040; A status * A chronicity^2 beta CI=-0.01-(-0.00), p = 0.018). Abbreviations: WMH; V = White Matter Hyperintensities, A = Amyloid PET, TICV = Total Intracranial Volume, PiB = Pittsburgh Compound-B, SILA = Sampled iterative local approximation.


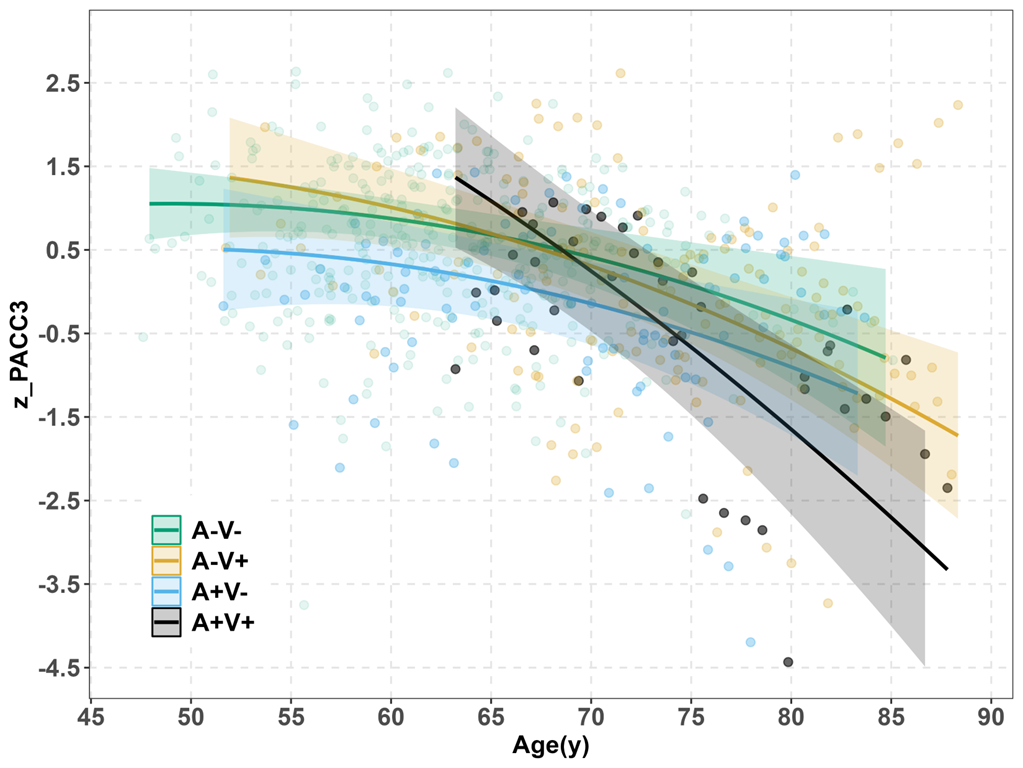


**Supplementary Fig. 9. Observed PACC3 z-scores (dots) in WADRC (Aim 4, n= 123) and simple slopes for A/V groups indicate the A+/V+ group declines fastest on average.**

Model was cognitive composite z-scores ~ c60_age * PiB/WMH groups + linear and quadratic c60_age + Sex + education + number of prior exposures + random person‐level intercepts and age slopes; c60_age indicates age centered at 60 (A-V+*age beta CI=-0.07 – 0.02, p = 0.298; A+V-*age beta CI=-0.05 – 0.04, p = 0.969; A+V+*age beta CI=-0.19 – -0.04, p = 0.003).

**Supplementary Table 1: Sample characteristics in WRAP and WADRC**

| **Variables** | **Aim 1 (Overall, n=877)** | **Aim 1**  **(V-; n=669)** | **Aim 1**  **(V+; n=208)** | **Aim 2 ad 3 (WRAP; n = 240)** | **Aim 4 (WADRC; n=123)** | **p-value** |
| --- | --- | --- | --- | --- | --- | --- |
| **Age at baseline WMH, mean(sd)** | 65.36 (8.49) | 63.25 (7.42) | 72.17 (8.12) | 67.16 (6.78) | 65.78 (8.49) | 0.094 |
| **Age at last WMH** | 67.26 (8.14) | 65.57 (7.29) | 73.87 (7.93) | 69.17 (6.89) | 68.42 (8.41) | 0.366 |
| **Age at last PiB** |  |  |  | 68.72 (6.72) | 68.22 (8.29) | 0.534 |
| **PiB Last Age - WMH Last Age** |  |  |  | -0.45 (1.59) | -0.21 (1.91) | 0.199 |
| **Female, n(%)** | 566 (64.5) | 429 (64.1) | 137 ( 65.9) | 165 (68.8) | 88 (71.5) | 0.669 |
| **Family history, n(%)** | 209 (23.9) | 172 (25.8) | 37 ( 17.8) | 170 (71.1) | 60 ( 66.7) | 0.021 |
| **White/Caucasian, n(%)** | 702 (84.5) | 543 (85.5) | 159 ( 81.1) | 226 (94.6) | 102 ( 82.9) | 0.001 |
| **College degree, n(%)** | 598 (68.2) | 472 (70.6) | 126 ( 60.6) | 166 (69.5) | 86 ( 69.9) | 0.999 |
| **WRAP, n(%)** | 272 (31.0) | 221 (33.0) | 51 ( 24.5) |  |  |  |
| **WRAT3 reading score** |  |  |  | 106.85 (8.98) |  |  |
| ***APOE* e4 count** |  |  |  |  |  | 0.994 |
| **0** | 468 (60.6) | 346 (59.1) | 122 ( 65.2) | 137 (59.8) | 70 (59.3) |  |
| **1** | 256 (33.2) | 201 (34.4) | 55 ( 29.4) | 77 (33.6) | 40 (33.9) |  |
| **2** | 48 ( 6.2) | 38 ( 6.5) | 10 ( 5.3) | 15 ( 6.6) | 8 ( 6.8) |  |
| **Last cognitive status, n(%)** |  |  |  |  |  | 0.651 |
| **CU** | 717 (81.8) | 591 (88.3) | 126 ( 60.6) | 211 (92.6) | 116 (94.3) |  |
| **MCI** | 71 ( 8.1) | 37 ( 5.5) | 34 ( 16.3) | 13 ( 5.7) | 7 ( 5.7) |  |
| Impaired-Other | 22 ( 2.5) | 17 ( 2.5) | 5 ( 2.4) | 0 ( 0.0) | 0 ( 0.0) |  |
| **Dementia** | 67 ( 7.6) | 24 ( 3.6) | 43 ( 20.7) | 4 ( 1.8) | 0 ( 0.0) |  |
| **Biomarker groups** |  |  |  |  |  | 0.202 |
| **A-V-** |  |  |  | 135 ( 56.2) | 76 (61.8) |  |
| **A-V+** |  |  |  | 37 ( 15.4) | 24 (19.5) |  |
| **A+V-** |  |  |  | 52 ( 21.7) | 16 (13.0) |  |
| **A+V+** |  |  |  | 16 ( 6.7) | 7 ( 5.7) |  |

Note: WRAT3 reading score is not available in WADRC. The groups V+- in aim 1 were created using the first WMH scan.

Abbreviations: WRAP = Wisconsin Registry for Alzheimer’s Prevention, WADRC = Wisconsin Alzheimer’s Disease Research Center, WMH = White Matter Hyperintensities, PiB = Pittsburgh Compound-B, WRAT3 = Wide Range Achievement Test-III Reading Recognition subtest, CU = Cognitive Unimpaired, MCI = Mild Cognitive Impaired, A = Amyloid, V = Cerebrovascular.

**Supplementary Table 2: WMH mixed effects output aim 1**

|  | Model 1 (Age) | | | Model 2 (V chronicity) | | |
| --- | --- | --- | --- | --- | --- | --- |
| *Predictors* | *Estimates* | *CI* | *p* | *Estimates* | *CI* | *p* |
| (Intercept) | 0.39 | 0.35 – 0.44 | **<0.001** | 0.97 | 0.94 – 1.00 | **<0.001** |
| C65age | 0.02 | 0.01 – 0.02 | **<0.001** |  |  |  |
| Baseline V+ | 1.21 | 1.14 – 1.29 | **<0.001** | 0.18 | 0.14 – 0.22 | **<0.001** |
| C65age^2 | 0.00 | 0.00 – 0.00 | **<0.001** |  |  |  |
| Female | -0.03 | -0.08 – 0.02 | 0.270 | 0.01 | -0.01 – 0.03 | 0.267 |
| C65age × Baseline V+ | 0.01 | 0.01 – 0.02 | **0.001** |  |  |  |
| V chronicity |  |  |  | 0.04 | 0.04 – 0.05 | **<0.001** |
| V chronicity^2 |  |  |  | 0.00 | 0.00 – 0.00 | **<0.001** |
| V chronicity × Baseline V+ |  |  |  | 0.06 | 0.05 – 0.06 | **<0.001** |
| **Random Effects** | | | | | | |
| σ^2^ | 0.02 | | | 0.02 | | |
| τ_00_ | 0.14 _reggieid_ | | | 0.01 _reggieid_ | | |
| ICC | 0.89 | | | 0.36 | | |
| N | 877 _reggieid_ | | | 877 _reggieid_ | | |
| Observations | 1572 | | | 1572 | | |
| Marginal R^2^ / Conditional R^2^ | 0.725 / 0.970 | | | 0.953 / 0.970 | | |
| AICc | 443.0 | | | -1405.4 | | |

Note: The full model is log(WMH+1)~ Time*Baseline V group+Time^2*Baseline V group + gender + random effects. Time is age centered at 65 in model 1, V chronicity=0 in model 2, separately. Non-significant interaction was removed from the full model.

**Supplementary Table 3: Chi‐square test of association between A and V positivity at last scan**

| **Cohort** |  | **V-** | **V+** | **p value** |
| --- | --- | --- | --- | --- |
| **WRAP** | **A-** | 135 | 37 | 0.86 |
|  | **A+** | 52 | 16 |  |
| **ADRC** | **A-** | 76 | 24 | 0.71 |
|  | **A+** | 16 | 7 |  |

**Supplementary Table 4: Aim 2a WMH mixed effects output for each of the four time operationalizations**

|  | **Model 1 (Time = Age)** | | | **Model 2 (Time = V+ chronicity)** | | | **Model 3 ((Time = Time since baseline))** | | | **Model 4 (Time = A+ chronicity)** | | |
| --- | --- | --- | --- | --- | --- | --- | --- | --- | --- | --- | --- | --- |
| *Predictors* | *Estimates* | *CI* | *p* | *Estimates* | *CI* | *p* | *Estimates* | *CI* | *p* | *Estimates* | *CI* | *p* |
| (Intercept) | 0.56 | 0.47 – 0.66 | **<0.001** | 1.20 | 1.16 – 1.23 | **<0.001** | 0.58 | 0.49 – 0.67 | **<0.001** | 0.79 | 0.64 – 0.94 | **<0.001** |
| PET A+ [A- = reference] | -0.10 | -0.30 – 0.11 | 0.358 | -0.01 | -0.06 – 0.04 | 0.726 | -0.02 | -0.25 – 0.21 | 0.844 | -0.54 | -0.81 – -0.27 | **<0.001** |
| c65_age [=Age (centered at 65)] | 0.02 | 0.01 – 0.03 | **<0.001** |  |  |  |  |  |  |  |  |  |
| c65_age^2 | 0.00 | -0.00 – 0.00 | 0.064 |  |  |  |  |  |  |  |  |  |
| c65_age * PET [PET A+] | -0.00 | -0.03 – 0.02 | 0.861 |  |  |  |  |  |  |  |  |  |
| PET [PET A+] * c65_age^2 | 0.00 | 0.00 – 0.00 | **0.011** |  |  |  |  |  |  |  |  |  |
| Baseline WMH age |  |  |  | 0.00 | 0.00 – 0.01 | **0.020** | 0.04 | 0.03 – 0.05 | **<0.001** | 0.04 | 0.02 – 0.05 | **<0.001** |
| V chronicity [=years since V+ onset] |  |  |  | 0.08 | 0.07 – 0.08 | **<0.001** |  |  |  |  |  |  |
| V chronicity^2 |  |  |  | 0.00 | 0.00 – 0.00 | **<0.001** |  |  |  |  |  |  |
| Time since first WMH scan [years] |  |  |  |  |  |  | 0.01 | 0.00 – 0.02 | **0.027** |  |  |  |
| Baseline WMH age* Time since first WMH scan |  |  |  |  |  |  | 0.00 | 0.00 – 0.00 | **0.018** |  |  |  |
| Baseline WMH age* PET A+ |  |  |  |  |  |  | -0.01 | -0.04 – 0.02 | 0.519 |  |  |  |
| Time since first WMH scan * PET A+ |  |  |  |  |  |  | 0.02 | -0.01 – 0.04 | 0.201 |  |  |  |
| (Baseline WMH age * Time since first WMH scan) * PET A+ |  |  |  |  |  |  | 0.00 | 0.00 – 0.01 | **0.015** |  |  |  |
| PET A+ Chronicity |  |  |  |  |  |  |  |  |  | 0.02 | 0.01 – 0.03 | **<0.001** |
| PET A+ Chronicity^2 |  |  |  |  |  |  |  |  |  | 0.00 | 0.00 – 0.00 | **<0.001** |
| **Random Effects** | | | | | | | | | | | | |
| σ^2^ | 0.02 | | | 0.02 | | | 0.02 | | | 0.02 | | |
| τ_00_ | 0.37 _reggieid_ | | | 0.02 _reggieid_ | | | 0.35 _reggieid_ | | | 0.37 _reggieid_ | | |
| ICC | 0.96 | | | 0.51 | | | 0.95 | | | 0.95 | | |
| N | 240 _reggieid_ | | | 240 _reggieid_ | | | 240 _reggieid_ | | | 240 _reggieid_ | | |
| Observations | 457 | | | 457 | | | 457 | | | 457 | | |
| Marginal R^2^ / Conditional R^2^ | 0.087 / 0.960 | | | 0.925 / 0.963 | | | 0.157 / 0.962 | | | 0.178 / 0.962 | | |
| AICc | 317.8 | | | -344.2 | | | 307.2 | | | 331.8 | | |

Note: Time operationalization, by model: 1) Age (centered at age 65); 2) V+ Chronicity (estimated years since or until V+ onset); 3) Time (years) since baseline scan*baseline WMH age; 4) PET A+ Chronicity. For models 2 and 4, the PET A+*Chronicity interactions allow testing whether estimated annual change in WMH differs between those who are PET A+ and A-. The model output in the table is the log-transformed.

**Supplementary Table 5 Aim 2a Sensitivity Analyses: WMH mixed effects output for PiB groups in two cohorts.**

|  | **A- in WRAP** | | | **A+ in WRAP** | | | **A- in WADRC** | | | **A+ in WADRC** | | |
| --- | --- | --- | --- | --- | --- | --- | --- | --- | --- | --- | --- | --- |
| *Predictors* | *Estimates* | *CI* | *p* | *Estimates* | *CI* | *p* | *Estimates* | *CI* | *p* | *Estimates* | *CI* | *p* |
| (Intercept) | 1.19 | 1.15 – 1.22 | **<0.001** | 1.23 | 1.15 – 1.31 | **<0.001** | 1.19 | 1.14 – 1.24 | **<0.001** | 1.09 | 0.83 – 1.34 | **<0.001** |
| Baseline WMH age | 0.01 | 0.00 – 0.01 | **0.004** | -0.00 | -0.01 – 0.01 | 0.618 | 0.00 | -0.00 – 0.01 | 0.442 | 0.01 | -0.01 – 0.03 | 0.265 |
| V chronicity | 0.07 | 0.07 – 0.08 | **<0.001** | 0.08 | 0.07 – 0.09 | **<0.001** | 0.07 | 0.07 – 0.08 | **<0.001** | 0.07 | 0.06 – 0.08 | **<0.001** |
| V chronicity ^2 | 0.00 | 0.00 – 0.00 | **<0.001** | 0.00 | 0.00 – 0.00 | **<0.001** | 0.00 | 0.00 – 0.00 | **<0.001** | 0.00 | 0.00 – 0.00 | **<0.001** |
| **Random Effects** | | | | | | | | | | | | |
| σ^2^ | 0.02 | | | 0.01 | | | 0.02 | | | 0.02 | | |
| τ_00_ | 0.02 _reggieid_ | | | 0.02 _reggieid_ | | | 0.02 _reggieid_ | | | 0.07 _reggieid_ | | |
| ICC | 0.48 | | | 0.59 | | | 0.50 | | | 0.74 | | |
| N | 184 _reggieid_ | | | 56 _reggieid_ | | | 104 _reggieid_ | | | 19 _reggieid_ | | |
| Observations | 347 | | | 110 | | | 241 | | | 39 | | |
| Marginal R^2^ / Conditional R^2^ | 0.921 / 0.959 | | | 0.936 / 0.974 | | | 0.900 / 0.950 | | | 0.911 / 0.977 | | |

**Note:** The model output for WMH in the table is the log-transformed.

**Supplementary Table 6: Aim 2b PiB mixed effects output for each of the four time operationalizations**

|  | **Model 1 (Time = Age)** | | | **Model 2 (Time = A+ chronicity)** | | | **Model 3 ((Time = Time since baseline))** | | | **Model 4 (Time = V+ chronicity)** | | |
| --- | --- | --- | --- | --- | --- | --- | --- | --- | --- | --- | --- | --- |
| *Predictors* | *Estimates* | *CI* | *p* | *Estimates* | *CI* | *p* | *Estimates* | *CI* | *p* | *Estimates* | *CI* | *p* |
| (Intercept) | 1.14 | 1.11 – 1.16 | **<0.001** | 1.23 | 1.22 – 1.24 | **<0.001** | 1.14 | 1.11 – 1.16 | **<0.001** | 1.22 | 1.18 – 1.26 | **<0.001** |
| V+ [V- = reference] | 0.05 | -0.03 – 0.12 | 0.220 | 0.02 | -0.00 – 0.04 | 0.125 | 0.05 | -0.03 – 0.13 | 0.227 | -0.03 | -0.12 – 0.05 | 0.435 |
| c65_age [=Age (centered at 65)] | 0.01 | 0.00 – 0.01 | **<0.001** |  |  |  |  |  |  |  |  |  |
| Baseline PiB PET age |  |  |  | 0.0005 | -0.0005 – 0.0015 | 0.318 | 0.005 | 0.001 – 0.009 | **0.011** | 0.003 | -0.001 – 0.007 | 0.142 |
| PET A+ Chronicity |  |  |  | 0.02 | 0.02 – 0.02 | **<0.001** |  |  |  |  |  |  |
| PET A+ Chronicity^2 |  |  |  | 0.0003 | 0.0003 – 0.0003 | **<0.001** |  |  |  |  |  |  |
| Time since first PiB PET |  |  |  |  |  |  | 0.007 | 0.005 – 0.009 | **<0.001** |  |  |  |
| Baseline PiB PET age × Time since first PiB PET |  |  |  |  |  |  | 0.0003 | 0.0001 – 0.0006 | **0.015** |  |  |  |
| V chronicity |  |  |  |  |  |  |  |  |  | 0.003 | 0.002 – 0.004 | **<0.001** |
| **Random Effects** | | | | | | | | | | | | |
| σ^2^ | 0.00 | | | 0.00 | | | 0.00 | | | 0.00 | | |
| τ_00_ | 0.04 _reggieid_ | | | 0.00 _reggieid_ | | | 0.04 _reggieid_ | | | 0.04 _reggieid_ | | |
| ICC | 0.89 | | | 0.39 | | | 0.90 | | | 0.90 | | |
| N | 240 _reggieid_ | | | 240 _reggieid_ | | | 240 _reggieid_ | | | 240 _reggieid_ | | |
| Observations | 638 | | | 638 | | | 638 | | | 638 | | |
| Marginal R^2^ / Conditional R^2^ | 0.049 / 0.899 | | | 0.912 / 0.946 | | | 0.053 / 0.901 | | | 0.084 / 0.906 | | |
| AICc | -935.8 | | | -1839.1 | | | -937.7 | | | -910.2 | | |

Note: Time operationalization, by model: 1) Age (centered at age 65); 2) A+ Chronicity (estimated years since or until A+ onset); 3) Time (years) since baseline scan*baseline PiB PET age; 4) V+ Chronicity. For models 2 and 4, the V+*Chronicity interactions allow testing whether estimated annual change in PiB differs between those who are V+ and V-.

**Supplementary Table 7 Aim 2b Sensitivity Analyses: PiB mixed effects output for WMH groups in two cohorts.**

|  | **V- in WRAP** | | | **V+ in WRAP** | | | **V- in WADRC** | | | **V+ in WADRC** | | |
| --- | --- | --- | --- | --- | --- | --- | --- | --- | --- | --- | --- | --- |
| *Predictors* | *Estimates* | *CI* | *p* | *Estimates* | *CI* | *p* | *Estimates* | *CI* | *p* | *Estimates* | *CI* | *p* |
| (Intercept) | 1.23 | 1.22 – 1.24 | **<0.001** | 1.26 | 1.21 – 1.31 | **<0.001** | 1.21 | 1.20 – 1.23 | **<0.001** | 1.20 | 1.16 – 1.23 | **<0.001** |
| Baseline PiB PET age | 0.00 | -0.00 – 0.00 | 0.240 | -0.00 | -0.01 – 0.00 | 0.953 | -0.00 | -0.00 – 0.00 | 0.944 | -0.00 | -0.00 – 0.00 | 1.000 |
| PiB chronicity | 0.02 | 0.02 – 0.02 | **<0.001** | 0.02 | 0.02 – 0.02 | **<0.001** | 0.02 | 0.02 – 0.02 | **<0.001** | 0.02 | 0.02 – 0.02 | **<0.001** |
| PiB chronicity ^2 | 0.00 | 0.00 – 0.00 | **<0.001** | 0.00 | 0.00 – 0.00 | **<0.001** | 0.00 | 0.00 – 0.00 | **<0.001** | 0.00 | 0.00 – 0.00 | **<0.001** |
| **Random Effects** | | | | | | | | | | | | |
| σ^2^ | 0.00 | | | 0.01 | | | 0.00 | | | 0.00 | | |
| τ_00_ | 0.00 _reggieid_ | | | 0.00 _reggieid_ | | | 0.00 _reggieid_ | | | 0.00 _reggieid_ | | |
| ICC | 0.44 | | | 0.12 | | | 0.19 | | | 0.00 | | |
| N | 209 _reggieid_ | | | 31 _reggieid_ | | | 93 _reggieid_ | | | 32 _reggieid_ | | |
| Observations | 573 | | | 65 | | | 103 | | | 34 | | |
| Marginal R^2^ / Conditional R^2^ | 0.902 / 0.945 | | | 0.930 / 0.938 | | | 0.958 / 0.966 | | | 0.961 / 0.961 | | |

**Supplementary Table 8 Mixed effects output for four cognitive composites effects (Aim 3)**

|  | **PACC3** | | | **EF** | | | **Immediate Learning** | | | **Delayed Recall** | | |
| --- | --- | --- | --- | --- | --- | --- | --- | --- | --- | --- | --- | --- |
| *Predictors* | *Estimates* | *CI* | *p* | *Estimates* | *CI* | *p* | *Estimates* | *CI* | *p* | *Estimates* | *CI* | *p* |
| **Intercept** | -0.27 | -0.48 – -0.06 | **0.011** | -0.13 | -0.32 – 0.07 | 0.205 | -0.35 | -0.57 – -0.13 | **0.002** | -0.33 | -0.54 – -0.12 | **0.002** |
| **Male** | -0.68 | -0.90 – -0.45 | **<0.001** | -0.31 | -0.52 – -0.10 | **0.004** | -0.58 | -0.81 – -0.34 | **<0.001** | -0.49 | -0.71 – -0.27 | **<0.001** |
| **WRAT3 reading** | 0.04 | 0.02 – 0.05 | **<0.001** | 0.02 | 0.01 – 0.03 | **<0.001** | 0.04 | 0.03 – 0.05 | **<0.001** | 0.04 | 0.03 – 0.05 | **<0.001** |
| **Practice** | 0.14 | 0.09 – 0.18 | **<0.001** | 0.08 | 0.04 – 0.12 | **<0.001** | 0.21 | 0.16 – 0.26 | **<0.001** | 0.19 | 0.14 – 0.24 | **<0.001** |
| **c60age** | -0.05 | -0.07 – -0.03 | **<0.001** | -0.06 | -0.08 – -0.04 | **<0.001** | -0.05 | -0.07 – -0.03 | **<0.001** | -0.04 | -0.06 – -0.02 | **<0.001** |
| **c60age^2** | -0.00 | -0.00 – 0.00 | 0.472 | -0.00 | -0.00 – 0.00 | 0.382 | -0.00 | -0.00 – 0.00 | 0.728 | 0.00 | -0.00 – 0.00 | 0.965 |
| **A-&V+** | -0.05 | -0.37 – 0.28 | 0.786 | -0.01 | -0.31 – 0.29 | 0.960 | -0.16 | -0.51 – 0.19 | 0.362 | -0.06 | -0.40 – 0.27 | 0.719 |
| **A+&V-** | 0.32 | 0.06 – 0.59 | **0.015** | 0.27 | 0.02 – 0.51 | **0.034** | 0.30 | 0.02 – 0.57 | **0.036** | 0.31 | 0.05 – 0.57 | **0.022** |
| **A+&V+** | 0.13 | -0.33 – 0.60 | 0.572 | -0.27 | -0.70 – 0.15 | 0.201 | 0.11 | -0.38 – 0.60 | 0.659 | -0.06 | -0.53 – 0.41 | 0.806 |
| **c60age * A-&V+** | -0.00 | -0.05 – 0.04 | 0.828 | 0.01 | -0.02 – 0.04 | 0.394 | -0.02 | -0.06 – 0.03 | 0.420 | -0.02 | -0.07 – 0.03 | 0.393 |
| **c60age * A+&V-** | -0.03 | -0.06 – 0.00 | 0.072 | -0.02 | -0.04 – 0.00 | 0.121 | -0.03 | -0.06 – 0.01 | 0.113 | -0.03 | -0.06 – 0.01 | 0.131 |
| **c60age * A+&V+** | 0.03 | -0.04 – 0.09 | 0.387 | -0.01 | -0.06 – 0.03 | 0.592 | 0.02 | -0.05 – 0.09 | 0.599 | 0.04 | -0.03 – 0.11 | 0.273 |
| **c60age^2 *** **A-&V+** | 0.00 | -0.00 – 0.00 | 0.804 | -0.00 | -0.00 – 0.00 | 0.424 | 0.00 | -0.00 – 0.00 | 0.681 | -0.00 | -0.00 – 0.00 | 0.846 |
| **c60age^2 * A+&V-** | -0.00 | -0.01 – -0.00 | **0.027** | -0.00 | -0.00 – -0.00 | **0.002** | -0.00 | -0.01 – -0.00 | **0.009** | -0.00 | -0.01 – -0.00 | **0.003** |
| **c60age^2 * A+&V+** | -0.01 | -0.01 – -0.00 | **<0.001** | -0.00 | -0.01 – -0.00 | **0.019** | -0.01 | -0.01 – -0.00 | **<0.001** | -0.01 | -0.01 – -0.01 | **<0.001** |
| **Random Effects** |  |  |  |  |  |  |  |  |  |  |  |  |
| **σ^2^** | 0.20 | | | 0.11 | | | 0.22 | | | 0.22 | | |
| **τ_00_** | 0.49 _reggieid_ | | | 0.48 _reggieid_ | | | 0.56 _reggieid_ | | | 0.48 _reggieid_ | | |
| **τ_11_** | 0.00 _reggieid.c60age_ | | | 0.00 _reggieid.c60age_ | | | 0.00 _reggieid.c60age_ | | | 0.00 _reggieid.c60age_ | | |
| **ρ_01_** | 0.08 _reggieid_ | | | 0.43 _reggieid_ | | | 0.07 _reggieid_ | | | -0.02 _reggieid_ | | |
| **ICC** | 0.76 | | | 0.85 | | | 0.77 | | | 0.76 | | |
| **N** | 237 _reggieid_ | | | 234 _reggieid_ | | | 237 _reggieid_ | | | 237 _reggieid_ | | |
| Observations | 1046 | | | 998 | | | 1046 | | | 1046 | | |
| Marginal R^2^ / Conditional R^2^ | 0.309 / 0.834 | | | 0.287 / 0.889 | | | 0.279 / 0.831 | | | 0.255 / 0.825 | | |

Note: Cog ~ age*4group+age^2+gender+WRAT3+Practice+random

**Supplementary Table 9: Mixed effects output of PACC3 in WRAP in Aim 3 sensitivity analysis**

|  | **Model 1_2way** | | | **Model 2_3way** | | |
| --- | --- | --- | --- | --- | --- | --- |
| *Predictors* | *Estimates* | *CI* | *p* | *Estimates* | *CI* | *p* |
| **(Intercept)** | -0.27 | -0.48 – -0.06 | **0.011** | -0.18 | -0.35 – -0.00 | **0.044** |
| **c60age** | -0.05 | -0.07 – -0.03 | **<0.001** | -0.06 | -0.08 – -0.04 | **<0.001** |
| **A-&V+** | -0.05 | -0.37 – 0.28 | 0.786 |  |  |  |
| **A+&V-** | 0.32 | 0.06 – 0.59 | **0.015** |  |  |  |
| **A+&V+** | 0.13 | -0.33 – 0.60 | 0.572 |  |  |  |
| **c60age^2** | -0.00 | -0.00 – 0.00 | 0.472 | -0.00 | -0.00 – -0.00 | **0.005** |
| **Male** | -0.68 | -0.90 – -0.45 | **<0.001** | -0.66 | -0.89 – -0.44 | **<0.001** |
| **WRAT3 reading** | 0.04 | 0.02 – 0.05 | **<0.001** | 0.03 | 0.02 – 0.05 | **<0.001** |
| **Practice** | 0.14 | 0.09 – 0.18 | **<0.001** | 0.13 | 0.09 – 0.18 | **<0.001** |
| **c60age * A-V+** | -0.00 | -0.05 – 0.04 | 0.828 |  |  |  |
| **c60age * A+V-** | -0.03 | -0.06 – 0.00 | 0.072 |  |  |  |
| **c60age * A+V+** | 0.03 | -0.04 – 0.09 | 0.387 |  |  |  |
| **c60age^2 * A-V+** | 0.00 | -0.00 – 0.00 | 0.804 |  |  |  |
| **c60age^2 * A+V-** | -0.00 | -0.01 – -0.00 | **0.027** |  |  |  |
| **c60age^2 * A+V+** | -0.01 | -0.01 – -0.00 | **<0.001** |  |  |  |
| **PET A+ Chronicity** |  |  |  | 0.13 | 0.02 – 0.23 | **0.019** |
| **V chronicity** |  |  |  | -0.04 | -0.15 – 0.07 | 0.495 |
| **c60age × PET A+ Chronicity** |  |  |  | -0.01 | -0.03 – -0.00 | **0.028** |
| **c60age × V chronicity** |  |  |  | -0.01 | -0.02 – 0.00 | 0.126 |
| **PET A+ Chronicity × V chronicity** |  |  |  | 0.05 | -0.06 – 0.15 | 0.398 |
| **PET A+ Chronicity × c60age^2** |  |  |  | -0.00 | -0.00 – -0.00 | **0.003** |
| **(c60age × PET A+ Chronicity) × V chronicity** |  |  |  | -0.01 | -0.02 – -0.00 | **0.048** |
| **Random Effects** | | | | | | |
| σ^2^ | 0.20 | | | 0.20 | | |
| τ_00_ | 0.49 _reggieid_ | | | 0.51 _reggieid_ | | |
| τ_11_ | 0.00 _reggieid.c60age_ | | | 0.00 _reggieid.c60age_ | | |
| ρ_01_ | 0.08 _reggieid_ | | | 0.04 _reggieid_ | | |
| ICC | 0.76 | | | 0.76 | | |
| N | 237 _reggieid_ | | | 237 _reggieid_ | | |
| Observations | 1046 | | | 1046 | | |
| Marginal R^2^ / Conditional R^2^ | 0.309 / 0.834 | | | 0.308 / 0.832 | | |
| AICc | 2027.3 | | | 2027.5 | | |

**Supplementary Table 10 Aim 2a replication WMH mixed effects output in WADRC (Aim 4)**

|  | **Model 1 (Time = Age)** | | | **Model 2 (Time = V+ chronicity)** | | | **Model 3 ((Time = Time since baseline))** | | | **Model 4 (Time = A+ chronicity)** | | |
| --- | --- | --- | --- | --- | --- | --- | --- | --- | --- | --- | --- | --- |
| *Predictors* | *Estimates* | *CI* | *p* | *Estimates* | *CI* | *p* | *Estimates* | *CI* | *p* | *Estimates* | *CI* | *p* |
| (Intercept) | 0.58 | 0.46 – 0.70 | **<0.001** | 1.19 | 1.13 – 1.24 | **<0.001** | 0.70 | 0.59 – 0.82 | **<0.001** | 1.07 | 0.85 – 1.29 | **<0.001** |
| c65_age [=Age (centered at 65)] | 0.02 | 0.01 – 0.03 | **<0.001** |  |  |  |  |  |  |  |  |  |
| PET A+ [A- = reference] | 0.08 | -0.23 – 0.38 | 0.614 | -0.02 | -0.12 – 0.08 | 0.684 | -0.02 | -0.34 – 0.29 | 0.877 | -1.26 | -1.83 – -0.68 | **<0.001** |
| c65_age^2 | 0.00 | 0.00 – 0.00 | **<0.001** |  |  |  |  |  |  |  |  |  |
| Baseline WMH age |  |  |  | 0.00 | -0.00 – 0.01 | 0.169 | 0.05 | 0.03 – 0.06 | **<0.001** | 0.05 | 0.03 – 0.06 | **<0.001** |
| V chronicity [=years since V+ onset] |  |  |  | 0.07 | 0.07 – 0.07 | **<0.001** |  |  |  |  |  |  |
| V chronicity^2 |  |  |  | 0.00 | 0.00 – 0.00 | **<0.001** |  |  |  |  |  |  |
| Time since first WMH scan [years] |  |  |  |  |  |  | 0.02 | 0.01 – 0.03 | **<0.001** |  |  |  |
| Baseline WMH age* Time since first WMH scan |  |  |  |  |  |  | 0.00 | 0.00 – 0.00 | **<0.001** |  |  |  |
| A+ Chronicity |  |  |  |  |  |  |  |  |  | 0.04 | 0.02 – 0.06 | **0.001** |
| A+ Chronicity^2 |  |  |  |  |  |  |  |  |  | 0.00 | 0.00 – 0.00 | **0.011** |
| A+ Chronicity* A+ |  |  |  |  |  |  |  |  |  | 0.08 | 0.00 – 0.16 | **0.040** |
| A+ Chronicity^2* A+ |  |  |  |  |  |  |  |  |  | -0.00 | -0.01 – -0.00 | **0.018** |
| **Random Effects** | | | | | | | | | | | | |
| σ^2^ | 0.02 | | | 0.02 | | | 0.02 | | | 0.02 | | |
| τ_00_ | 0.36 _reggieid_ | | | 0.03 _reggieid_ | | | 0.35 _reggieid_ | | | 0.36 _reggieid_ | | |
| ICC | 0.96 | | | 0.57 | | | 0.96 | | | 0.96 | | |
| N | 123 _reggieid_ | | | 123 _reggieid_ | | | 123 _reggieid_ | | | 123 _reggieid_ | | |
| Observations | 280 | | | 280 | | | 280 | | | 280 | | |
| Marginal R^2^ / Conditional R^2^ | 0.259 / 0.969 | | | 0.910 / 0.962 | | | 0.338 / 0.972 | | | 0.354 / 0.971 | | |
| AICc | 118.5 | | | -140.7 | | | 116.7 | | | 134.2 | | |

Note: Time operationalization, by model: 1) Age (centered at age 65); 2) V+ Chronicity (estimated years since or until V+ onset); 3) Time (years) since baseline scan*baseline WMH age; 4) PET A+ Chronicity. For models 2 and 4, the PET A+*Chronicity interactions allow testing whether estimated annual change in WMH differs between those who are PET A+ and A-. The model output for WMH in the table is the log-transformed.

**Supplementary Table 11 Mixed effects output of PACC3 in WADRC (Aim 4)**

|  | **PACC3** | | |
| --- | --- | --- | --- |
| *Predictors* | *Estimates* | *CI* | *p* |
| **(Intercept)** | 0.57 | 0.34 – 0.80 | **<0.001** |
| **c60age** | -0.03 | -0.06 – -0.01 | **0.018** |
| **A-&V+** | 0.13 | -0.34 – 0.61 | 0.582 |
| **A+&V-** | -0.55 | -1.03 – -0.06 | **0.027** |
| **A+&V+** | 0.98 | -0.01 – 1.96 | 0.052 |
| **c60age^2** | -0.00 | -0.00 – -0.00 | **0.039** |
| **Male** | -0.12 | -0.44 – 0.21 | 0.479 |
| **Practice** | 0.11 | 0.07 – 0.15 | **<0.001** |
| **No bachelor’s degree** | -0.56 | -0.87 – -0.25 | **<0.001** |
| **c60age * A-V+** | -0.02 | -0.07 – 0.02 | 0.298 |
| **c60age * A+V-** | -0.00 | -0.05 – 0.04 | 0.969 |
| **c60age * A+V+** | -0.11 | -0.19 – -0.04 | **0.003** |
| **Random Effects** | | | |
| σ^2^ | 0.25 | | |
| τ_00_ _reggieid_ | 0.46 | | |
| τ_11_ _reggieid.c60age_ | 0.00 | | |
| ρ_01_ _reggieid_ | 0.34 | | |
| ICC | 0.76 | | |
| N _reggieid_ | 123 | | |
| Observations | 630 | | |
| Marginal R^2^ / Conditional R^2^ | 0.331 / 0.838 | | |

Note: Cog ~age*4group+age^2+gender+education+Practice+random effects
